# A Genome-Wide Association Study of Taste Liking in the Danish Population

**DOI:** 10.1016/j.tjnut.2025.06.001

**Published:** 2025-06-07

**Authors:** Sara Haydar, Camilla Cederbye Karlsson, Allan Linneberg, Line Lund Kårhus, Lars Ängquist, Oluf Pedersen, Wender Bredie, Torben Hansen, Niels Grarup

**Affiliations:** 1 Novo Nordisk Foundation Center for Basic Metabolic Research, Faculty of Health and Medical Sciences, University of Copenhagen, Copenhagen, Denmark; 2 Center for Clinical Research and Prevention, Copenhagen University Hospital – Bispebjerg and Frederiksberg, Copenhagen, Denmark; 3 Department of Clinical Medicine, Faculty of Health and Medical Sciences, University of Copenhagen, Copenhagen, Denmark; 4 Center for Clinical Metabolic Research, Herlev–Gentofte Hospital, Copenhagen, Denmark; 5 Section for Food Design and Consumer Behavior, Department of Food Science, University of Copenhagen, Frederiksberg, Denmark

**Keywords:** liking, taste, GWAS, SNPs, Danish

## Abstract

**Background:**

Taste liking, a complex trait, plays an important role in food choice and eating behavior, thereby influencing risk of diet-related diseases.

**Objectives:**

This study aimed to identify novel loci that could explain differences in liking of 5 basic tastes, fat sensation, and 2 oral sensations, represented by several food items.

**Methods:**

Liking scores were derived using a newly developed taste liking questionnaire (TasteLQ), validated in the Danish population. We conducted a genome-wide association study (GWAS) of liking of 6 modalities (sweet, salty, sour, bitter-astringency, umami, and pungency) and 9 factors representing modality subgroups among 6,437 Danish adults. As a secondary analysis, GWASs of 44 single food items from TasteLQ were also undertaken.

**Results:**

We identified 1 genome-wide significant variant, rs170518 (minor allele frequency = 0.16), on chromosome 5, associated with liking of an umami factor characterized by glutamate-rich food items [*P* = 3.7 × 10^−8^, beta = 0.14 standard deviation (SD) (standard error (SE)) = 0.03]. When analyzing individual food items, 4 single nucleotide polymorphisms (SNPs) within 1 locus, annotated to the bitter taste receptor gene, *TAS2R38*, were associated with liking of bitter-tasting rocket salad. Finally, our data confirmed some of the previously associated genomic variants with taste perception, food liking, and intake.

**Conclusions:**

Although our findings provide insight into loci involved in taste liking, they remain preliminary and warrant additional validation due to lack of replication in an independent population and limited number of genome-wide significant associations.

## Introduction

Unhealthy dietary patterns have been linked to several cardiometabolic disorders, such as obesity, diabetes, and cardiovascular diseases [1]. Taste, a sensory method encompassing sweet, salty, sour, bitter, and umami, influences food liking and decision-making process underlying food choices, potentially impacting diet-related disease risk [2]. Food liking, a behavioral trait, emerges from complex interactions of environmental and genetic factors [3], showing higher heritability compared with food consumption [4].

Physiologically, taste perception is initiated when nutrient stimuli are detected by taste receptor cells, followed by signal transmission to the gustatory cortex [5]. Individual differences in taste perception with variations in taste receptor genes have been identified as components of human evolution [6]. Therefore, not surprisingly, there would be a strong link between taste perception, taste and food preference, and food intake [[7], [8], [9], [10]]. When studying this complex relationship, considering ethnic diversity and environmental factors is important, as perception and liking vary between populations [11]. One example is variation in the *TAS2R38* gene*,* encoding the bitter taste receptor, which alters bitter taste sensitivity and results in different bitter taste phenotypes [12]. The impact of bitter taste sensitivity level on preference and consumption of bitter-tasting foods, such as vegetables, remains inconsistent according to the literature [[13], [14], [15], [16]].

Several genome-wide association studies (GWASs) have identified loci associated with food liking and preferences, beyond genes encoding taste receptors [4,[17], [18], [19]]. For instance, variants in the fibroblast growth factor 21 (*FGF21*) gene were associated with higher liking and consumption of sweet foods [4,20]. The obesity-related *FTO* was also associated with sweet preference, potentially through a neural mechanism [21]. These previous GWASs focused on food liking without addressing the taste of the food as a central aspect of liking as a trait, whereas others focused on perception or liking of specific tastes [4,[22], [23], [24]]. Beyond examining the entire food matrix, where liking is influenced by taste, texture, and smell, our focus extends to elucidating the genetic factors contributing to taste liking.

This study primarily aimed to identify novel loci that explain differences in taste liking, using a newly developed and validated questionnaire to measure liking of taste and oral sensations represented by various food items. In this context, we present results from a GWAS of liking of 5 basic tastes (sweet, salty, sour, bitter, and umami), fat sensation, and 2 oral sensations (astringency and pungency) in the Danish adult population. As a secondary aim, we also explored whether a GWAS of individual food items representing these tastes and sensations could provide additional insights.

## Methods

### Study populations

Former participants from 5 Danish cohorts (DanFunD, Health2006, Health2008, Health2010, and Inter99) were invited to participate in the study. These cohorts were population based and included randomly sampled individuals from the background population [25,26]. A brief description of each cohort is presented in Supplemental Table 1. For this study, participants were re-contacted digitally by e-Boks (https://private.e-boks.com/danmark/en) in 2022 and invited to complete the Web-based validated taste liking questionnaire (TasteLQ) [27] and lifestyle-related questions, via the REDCap application after providing written digital informed consent.

### Taste-liking phenotypes

Taste-liking scores were calculated using the TasteLQ, which measures the recalled taste liking in the Danish adult population [27]. The TasteLQ includes 44 food items representing the basic taste modalities (sweet, salty, sour, bitter, and umami), fat sensation, as well as 2 oral sensations (pungency and astringency). Participants were asked to answer the question “*How much do you like the taste of [food item]?*” and rate their liking on a 0–100 visual analog scale anchored by “Do not like at all” and “Like very much” using a slider. Liking scores were calculated for 6 modalities (sweet, salty, sour, bitter-astringency, umami, and pungency) and 9 underlying factors (sweet-fatty items, naturally sweet items/fruits, sweeteners, dairy products, sour-low fat items, bitter-astringent beverages, other bitter items, savory-meat items, and other umami items) according to the taste-liking structure identified using factor analysis during the TasteLQ validation, as previously described [27]. Briefly, scores for each underlying factor were calculated by averaging scores of representative food items, whereas scores representing modalities were computed by averaging the total liking score of the corresponding underlying factors (Supplemental Figure 1).

### Covariates

The following covariates were collected by self-report from participants at the time of completing the TasteLQ: age (years), weight (kg), height (cm), and lifestyle factors (smoking, alcohol consumption, and physical activity). BMI (in kg/m^2^) was derived from self-reported weight and height. Smoking status was categorized into 4 groups: daily smokers, occasional smokers, ex-smokers, and never smokers [28]. Physical activity levels (active and inactive) were derived based on total weekly time spent on both commuting and leisure-time physical activity. Active status was defined as >225 min per week, whereas inactive status was defined as ≤225 min per week [28,29]. Alcohol consumption was categorized as “no or moderate” (≤6 units of alcohol per week for females and ≤12 units per week for males) and “high” (>6 units of alcohol per week for females and >12 units per week for males) [28].

### Statistical and computation methods

#### GWAS of taste liking

Participants were genotyped using Illumina HumanOmniExpress BeadChip (Omniexp24 and Omniexp24A). Genotypes were called using Genotyping module (version 1.9.4) in GenomeStudio software (version 2011.1; Illumina). Ethnic outliers, samples with a high level of genotype missingness, and sex mismatches were excluded. Before imputation, monomorphic variants and those deviating from the Hardy–Weinberg equilibrium (HWE) (*P* < 1 × 10^−5^) and with low call rates (<98%) were removed. Genotyped data were imputed with the Michigan imputation server using the Haplotype Reference Consortium reference panel. To reduce computational time, imputed SNPs with quality score (*R*^2^) < 0.8 were excluded, resulting in 10,688,504 variants for the final analysis.

The main GWAS included a combined data set from the 5 cohorts (*n* = 6,347) and was performed separately within the 6 modalities and underlying factors of the TasteLQ (Supplemental Figure 1) using rank-based inverse normal transformed liking scores to account for the nonnormal data distribution. The transformed scores present a mean of 0 and an SD of 1. We also performed GWASs of the 44 food items from the TasteLQ (Supplemental Figure 1). Analyses were carried out using RVTESTS (version 2.1.0) applying a linear mixed model regression and the score test under an additive genetic model [30]. To account for familial relatedness and population stratification, a kinship matrix based on pruned independent genotyped SNPs with minor allele frequency (MAF) > 0.01 was generated using the *vcf2kinship* tool and included in the analyses as a random effect. The basic model included age, age^2^, and sex as covariates. Another model was considered, further adjusting for BMI to address adiposity-related effects. Additionally, for loci with genome-wide significance, we performed sensitivity analyses, including smoking, alcohol consumption, and physical activity as covariates, to account for the effects of lifestyle factors. Individuals with missing phenotype data per taste modality, factor, or covariates were automatically removed from analyses by RVTESTS. Specifically, 2 individuals were excluded from all analyses. However, the number of exclusions varied for other GWASs: sour-dairy and sweet-fruit (*n* = 5), sweet-sweeteners (*n* = 25), umami-others (*n* = 39), and umami-savory (*n* = 8).

Results were filtered by including variants with MAF > 0.01 and HWE *P* > 1 × 10^−6^. Genomic inflation factor (lambda) was calculated, and quantile–quantile plots were generated using R package *qqman*. Manhattan plots were obtained using the *CMplot* R package. The genome-wide association significance threshold was defined at *P* < 5 × 10^−8^, whereas *P* < 1 × 10^−5^ was considered as a suggestive association. Variants were annotated to the closest gene using the SNPnexus v4 annotation tool (https://www.snp-nexus.org/v4/) and genome assembly GRCh37/hg19. Specifically, we reported the name of the gene to which the variant is overlapped or the nearest upstream and downstream genes when the variant is intergenic. Compliance with Human Genome Variation Society nomenclature was verified in VariantValidator (https://variantvalidator.org/).

#### Fine mapping

To identify independent loci among genome-wide significant SNPs in a locus, we performed a conditional analysis by including the lead SNP as covariate in the regression model. A locus was defined as the region ±500 kb around the SNP with the lowest *P* value. Fine mapping using the Sum of Single Effect model was performed as an alternative, to identify a credible set of causal variants [31]. Summary statistics were used as input, considering a 3 Mb window around the lead SNP, with the in-sample linkage disequilibrium (LD) matrix computed using PLINK 1.9 [32].

To further explore the role of the genome-wide significant variants identified in GWAS of underlying factors of TasteLQ, we performed a fine-scale haplotype mapping of the region surrounding the lead SNP. We first identified LD block structure using HAPLOVIEW [33], followed by haplotype reconstruction in each LD block in unrelated individuals (*n* = 5,964) using the PHASE program, which implements a Bayesian statistical method [34]. Haplotypes were then assigned to each individual as haplotype pairs. The sample of unrelated individuals was obtained by removing those with the lowest call rate of each related pair. The association of haplotype pairs with taste-liking scores was performed using a linear regression model adjusted for age, age^2^, and sex.

Of note, the 3 variants of *TAS2R38* gene (rs10246939, rs1726866, and rs713598), which were previously associated with bitter taste perception, were also included in the haplotype analysis to obtain an overview of the taste-liking phenotypes considering their combination [2,3]. This combination results in the *taster* phenotype with the haplotype PAV (proline–alanine–valine) and the *nontaster* phenotype with AVI (alanine–valine–isoleucine). Liking scores of modalities, underlying factors, and food items were compared across the different *TAS2R38* haplotype pairs using the Kruskal–Wallis test and the post hoc Wilcoxon rank sum test for pairwise comparison with Bonferroni adjustment. The association of liking scores with the *TAS2R38* haplotypes was evaluated using the haplo.score function in the R package *haplo.stats*, where posterior probabilities are used to compute the score statistics for association.

#### Investigation of previously associated taste-related SNPs

To confirm the credibility of our findings, we explored 28 SNPs in loci previously reported to be associated with taste perception, food liking, and intake in our main GWAS data. Inclusion was based on known loci with biological implications, such as taste receptor genes (e.g., bitter and sweet taste receptors), as well as loci previously reported in well-powered GWAS studies. Details about the 28 SNPs are found in Supplemental Table 2.

#### Gene expression and enrichment analysis

Tissue expression analysis was performed using genes that overlap variants associated with taste-liking modalities in the main GWAS at both suggestive (*P* < 1 × 10^−5^) and genome-wide (*P* < 5 × 10^−8^) significance thresholds. Suggestive variants were included in these analyses as prior studies indicate their potential biological role [35]. The Web tool functional mapping and annotation of GWAS (FUMA) (https://fuma.ctglab.nl/) with data from GTEx v8 across 30 general and 54 specific tissue types was used. Tissue specificity was determined for the input genes using a hypergeometric test with Bonferroni correction. To gain insight into biological pathways associated with these genes, we used the function enrichment tool in the GeneNetwork v2.0 (https://www.genenetwork.nl/), which predicts enriched pathways for gene sets.

#### Functional analysis

To examine the functional role of identified genome-wide significant variants, we used HaploReg v4.2 (https://pubs.broadinstitute.org/mammals/haploreg/haploreg.php), GTEx v8 portal (https://gtexportal.org/home/), and Open Targets variant-to-gene pipeline (https://genetics.opentargets.org/). This included prioritizing potential functional genes implicated by these variants and exploring their associations with gene expression, splicing, and protein levels through quantitative trait loci (expression quantitative trait loci (eQTL), splicing quantitative trait loci, and protein quantitative trait loci (pQTL)) analyses.

#### Phenome-wide association study

We used the GWAS Atlas (https://atlas.ctglab.nl/PheWAS) to explore pleiotropic effects of the genome-wide significant variants, querying against 4,756 traits while applying a Bonferroni correction (*P* = 0.05/4,756 = 1.1 × 10^−5^). The Open GWAS database (https://gwas.mrcieu.ac.uk/) was also consulted.

#### SNP-based heritability of taste liking

SNP-based heritability estimates of TasteLQ taste modalities and underlying factors were calculated using the Genomic-wide complex trait analysis (GCTA) - Genomic-Relatedness-Matrix Restricted Maximum Likelihood (GREML) method [36]. The same covariates as used in the basic model were applied. The genetic relationship matrix was estimated using imputed SNPs with imputation score *R*^2^ > 0.8, HWE *P* > 1 × 10^−6^, and MAF > 0.01. To account for cryptic relatedness, a cutoff value of 0.05 for estimated relatedness was used.

#### Genetic correlations

SNP-based bivariate genetic correlation analyses were performed among taste modalities using GWAS summary statistics. To investigate the shared genetic architecture between taste liking and other phenotypes, genetic correlations were also estimated between taste modalities, underlying factors and 33 phenotypes focusing on diet and lifestyle factors, biochemical measurements, education, psychiatric traits, food liking, and brain volume (phenotypes are presented in Supplemental Table 3). Of note, other metabolic or biochemical phenotypes were not investigated in this study. LD score regression with LDSC (version 1.0.1) and HapMap3 SNP filtering were used [37]. Precomputed LD score reference files based on 1000 Genome Project European data were considered. *P* values were adjusted using false discovery rates, and genetic correlations were visualized with the R package *corrplot* [38].

## Results

### Study population and taste-liking phenotypes

This study included 6,347 Danish individuals (57.3% females) with a median (IQR) age of 65 (57–72) y at the time of TasteLQ completion. Participant characteristics and liking scores are shown in Table 1. Within the population, the salty taste modality was the most liked with a median (IQR) liking score of 74.6 (62.8–84.6), whereas the sour modality had the lowest score of 55.7 (46.0–65.8). Regarding lifestyle factors, 62.7% of participants reported alcohol consumption in the “no or moderate” category, and 61.7% were physically active. The majority of participants were “never smokers” (47.8%) or “ex-smokers” (42.9%).

**TABLE 1:** Characteristics of the study population and taste liking scores.

Variables^1^	Total (*n* = 6347)
Sex (% females)	57.3
Age (y)^1^	65 (57–72)
BMI (kg/m^2^)	25.8 (23.4–29.0)
Sweet taste liking	71.3 (61.1–79.8)
Sweet-fatty items	72.3 (59.9–82.6)
Naturally sweet items/fruits	82.3 (69.7–91.7)
Sweeteners	63.0 (45.0–78.0)
Sour taste liking	55.8 (46.0–65.8)
Dairy products	63.7 (49.0–77.5)
Sour-low fat items	52.0 (41.0–62.8)
Salty taste liking	74.6 (62.8–84.6)
Umami taste liking	74.3 (64.8–82.8)
Savory-meat items	78.5 (67.6–87.3)
Other umami items	70.5 (55.0–82.0)
Bitter-astringency taste liking	68.0 (57.1–77.6)
Bitter-astringent beverages	69.5 (54.3–81.3)
Other bitter-astringent items	68.3 (56.3–79.8)
Pungency taste liking	60.0 (46.8–72.0)
Alcohol consumption (*n*, %)^2^	
No or moderate	3977 (62.7)
High	1853 (29.2)
Physical activity (*n*, %)	
Active	3918 (61.7)
Inactive	2429 (38.3)
Smoking (*n*, %)	
Daily smokers	463 (7.3)
Occasional smokers	127 (2.0)
Ex-smokers	2727 (42.9)
Never	3030 (47.8)

Data are presented as median (IQR) or percentage (%). Liking scores (0–100) are reported by modalities and underlying factors.

1 Data were collected at the time of completion of the TasteLQ.

2 Missing data (*n* = 517).

### GWAS of taste-liking phenotypes

**FIGURE 1:**
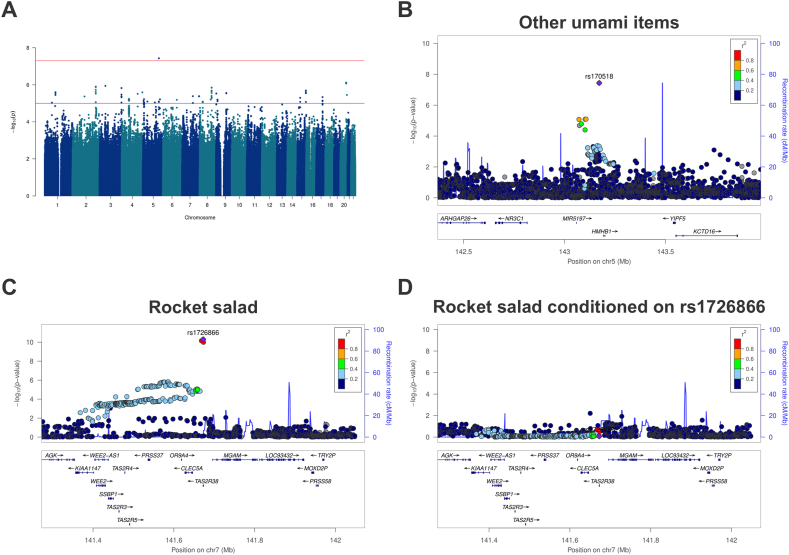
Manhattan and regional plots of genome-wide significant signals in the study population (*n* = 6,347). (A) Manhattan plot for the GWAS of other umami items factor liking, (B) regional plot of the locus associated with other umami items factor liking, (C) regional plot of the locus associated with rocket salad liking, and (D) regional plot of the locus associated with rocket salad liking after conditional analysis. In (A), the blue horizontal line represents the genome-wide suggestive threshold (*P* < 1 × 10^−5^) and the red line represents the genome-wide significant threshold (*P* < 5 × 10^−8^). Results represent the basic model using the rank-based inverse normal transformed liking scores and adjusted for age, age^2^, sex. In (B, C, and D), the *x*-axis shows the genomic position (GRCh37/hg19) and the left-hand *y*-axis the −log10(*P* value). Blue peaks correspond to the recombination rate (cM/Mb) quantified on the right-hand *y*-axis. Purple diamonds represent genome-wide significant variants. Color codes are interpreted as degree of linkage disequilibrium with the genome-wide variant. The bottom panel displays the genes in the region and the arrows represent the direction of transcription. cM, centimorgan; Mb, megabase.

No evidence of test statistics inflation was detected across modalities and underlying factors (lambda: 1.00–1.02) (Supplemental Figure 2). The most significant association was identified within the other umami-items factor, composed of parmesan cheese and soy sauce, where we found a genome-wide significant signal, at rs170518 (MAF_C-allele_ = 0.16), which was associated with a higher liking score [T allele; *P* = 3.7 × 10^−8^, beta = 0.14 SD (SE = 0.03)] (Table 2; Figure 1A). Liking scores for other umami-items factor in the population according to the rs170518 genotypes are shown in Supplemental Figure 3. This variant was an imputed SNP (imputation score *R*^2^ = 0.92) and annotated to an lncRNA, *CTB-57H20.1*, on chromosome 5 (Figure 1B). However, VariantValidator indicated no transcript overlaps with this variant. The regional plot showed that rs170518 was in low LD (*R*^2^ < 0.8) with 3 suggestively associated SNPs (Figure 1B). Sensitivity analysis revealed that further adjustment for smoking or physical activity did not affect the statistical significance of the lead SNP. When adding alcohol consumption as a covariate, a slight reduction of the effect size was observed, whereas the association was no longer genome-wide significant [T allele; *P* = 3.4 × 10^−6^, beta = 0.12 SD (SE = 0.03)].

**TABLE 2:** Independent genome-wide significant variants associated with taste liking phenotypes.

Phenotype	SNP ID	Chr	Position	Effect allele	Other allele	EAF	Closest gene	Beta, SD	SE	*P* value
Other umami-items^1^	rs170518	5	143171581	T	C	0.843	*CTB-57H20.1*	0.140	0.026	3.7 × 10^−8^
Rocket salad^2^	rs1726866	7	141672705	A	G	0.577	*MGAM, TAS2R38*	0.115	0.017	5.1 × 10^−11^

Results are based on rank-based inverse normal transformed liking scores, adjusted for age, age^2^ and sex. The closest gene indicates the overlapped gene. Genomic locations are shown as GRCh37/hg19.

Chr, chromosome; Beta, effect size; EAF, effect allele frequency.

1 GWAS results of “other umami-items” factor.

2 GWAS results of the individual item “rocket salad.”

To further explore the credibility of the association of rs170518 with the other umami-items factor, we first performed fine-mapping, which showed that this SNP was the only potential causal variant in the region, with a posterior inclusion probability (PIP) of 0.95. Analysis was further completed by fine-scale haplotype mapping in 2 LD blocks defined in this region, the rs170518 being located in block 2. In block 1, 3 major haplotypes were identified (H1 42%, H3 33%, and H5 22%), whereas in block 2, another 3 haplotypes were identified (H1 59%, H2 25%, and H3 16%) (Supplemental Figure 4A, B). Haplotype analysis (excluding pairs with a frequency below 2%) revealed significant associations with liking scores for the other-umami factor. Carriers of haplotype pairs containing the lead SNP from block 2 showed both positive (**T**GCA/**T**ATT, *P* = 2.7 × 10^-2^) and negative (**T**GCA/**C**GCA, *P* = 3.5 × 10^-3^; **C**GCA/**C**GCA, *P* = 5.8 × 10^-4^) associations (Supplemental Table 4).

Several suggestive associations were detected across all the TasteLQ modalities and factors: 69 loci (sweet), 17 loci (salty), 54 loci (sour), 53 loci (bitter-astringency), 70 loci (umami), and 18 loci (pungency) (Supplemental Figure 5). Including BMI as a covariate did neither substantially change the number of significant SNPs nor the effect size estimates.

### GWAS of food items

Among the 44 single food items, only the analysis of rocket salad (also known as arugula or in Danish *rucola salat*) liking revealed 4 genome-wide significant SNPs in 1 locus (chromosome 7) annotated to *MGAM* and *TAS2R38* [39]. The lead SNP of the locus was rs1726866 [*P* = 5.1 × 10^−11^, beta = 0.12 SD (SE=0.017)] (Table 2), and conditional analysis showed that the remaining SNPs (rs4726481, rs10246939, and rs713598) were not independent of rs1726866 (Figure 1C, D). Further adjustment for lifestyle factors slightly decreased statistical significance of the independent SNP rs1726866, with no major change in effect size [*P* = 1.5 × 10^−10^, beta = 0.11 SD (SE=0.017)]. Haplotype analysis, encompassing the 3 SNPs (rs10246939, rs1726866, and rs713598), revealed that homozygous individuals with PAV/PAV haplotype pair displayed lower liking for rocket salad compared with individuals with the AVI/AVI haplotype pair (Supplemental Results and Supplemental Figure 6).

### Analysis of previously associated taste-related SNPs

Due to the limited genome-wide significant associations, we next explored in our data 28 variants previously reported to be associated with food liking or consumption and taste perception. This step was intended to verify the credibility of our findings and confirm that the low power could explain the lack of signals. Of these 28 variants, 9 SNPs showed an association with a TasteLQ-liking phenotype at *P* < 0.05. For instance, rs1260326 (C allele) in *GCKR* was associated with bitter-astringent beverages liking (including red wine, beer, tonic water, and coffee) (*P* = 7 × 10^−4^). The *FTO* variant rs10852521 (C allele), associated with increased honey liking in UK Biobank [4], was associated with higher sweet (*P* = 0.03) and sweet-fatty (*P* = 0.04) liking in our population. Variant rs2733520 in *TBC1D5*, associated with fatty/dairy liking in UK Biobank [4], was confirmed in the analysis of sour-dairy items factor (*P* = 0.01). Moreover, a stronger association was observed with the sour modality (*P* = 0.003). Considering umami taste liking, the G allele of *FGF21* variant, rs838133, associated with increased liking of glutamate and savory food [4]. In our population, it was likewise positively associated with other umami items factor liking (*P* = 0.007). Previous research has indicated a positive association between rs1726866 (A allele) within *TAS2R38* and perceptions of bitter taste and grapefruit liking [4,22]. From our data, we observed a positive association with the other bitter-astringency factor encompassing rocket salad, black tea, dark chocolate, and walnuts (*P* = 0.002). Lastly, rs4939727 (A allele) in *DCC* [4], linked to pungent-tasting food liking, was detected in our data (*P* = 0.05). These results are presented in Supplemental Table 5.

### Gene expression and enrichment analysis

Variants associated with the 6 modalities and underlying factors of the TasteLQ at genome wide and suggestive significance levels overlapped with a total of 171 genes, as annotated by the SNPnexus tool. These genes were used as input in the FUMA GENE2FUNC process. Tissue expression analysis revealed significant enrichment in the brain and specifically in the hypothalamus, frontal cortex, and nucleus accumbens (Supplemental Figure 7A, B). Of the 171 genes, 169 were identified in GeneNetwork and underwent pathway enrichment analysis. The most significantly enriched REACTOME pathways were related to the neuronal system, including transmission across chemical synapses, neurotransmitter receptors, postsynaptic signal transmission, gamma-aminobutyric acid synthesis, release, reuptake, and degradation. Pathways related to sensory perception, including olfactory signaling pathway and signal transduction, also including the effects of PIP2 hydrolysis, were similarly enriched (Supplemental Table 6). Results from Kyoto Encyclopedia of Genes and Genomes and Gene Ontology analyses showed similar patterns, confirming consistency across pathway databases (Supplemental Table 6).

### Functional annotation and phenome-wide association study

In Open Targets, the Nuclear Receptor Subfamily 3 Group C Member 1 (*NR3C1*) gene*,* located 356 kb from the umami-lead SNP rs170518, displayed the highest variant-to-gene score, followed by *HMHB1* (Histocompatibility Minor HB-1) and *YIPF5* (Yip1 domain family member 5) located 20 and 378 kb, respectively, from the variant. This score, indicating the potential functional gene associated with this variant, is based on various parameters, including physical distance, in silico functional prediction, QTLs, and chromatin interaction experiments (e.g., Promotor Capture Hi-C). Additionally, rs170518 has been reported to associate with *HMHB1* expression in whole blood (*P* = 3.2 × 10^−3^) based on eQTL in HaploReg derived from a study of 5,311 individuals [40]. This variant also alters regulatory binding motifs of several transcription factors, including DMRT5, Elf3, Foxd1, HDAC2, Irf, Mef2, PRDM1, STAT, TATA, and p300.

From the Open GWAS database, rs170518 has been reported to be a pQTL of the glucocorticoid receptor encoded by *NR3C1* in a plasma proteome analysis among 3,301 healthy Europeans [T allele; *P* = 9.3 × 10^−4^; beta (SE): 0.12 (0.04)] [41]. The variant also showed 158 associations with other traits, including psychiatric, metabolic, and nutritional traits in the GWAS Atlas (Supplemental Figure 8), although not significant after Bonferroni correction. Among the nutritional traits were cereal intake, salad/raw vegetable intake, type of milk used (full cream), and oily fish intake.

### SNP-based heritability

**FIGURE 2:**
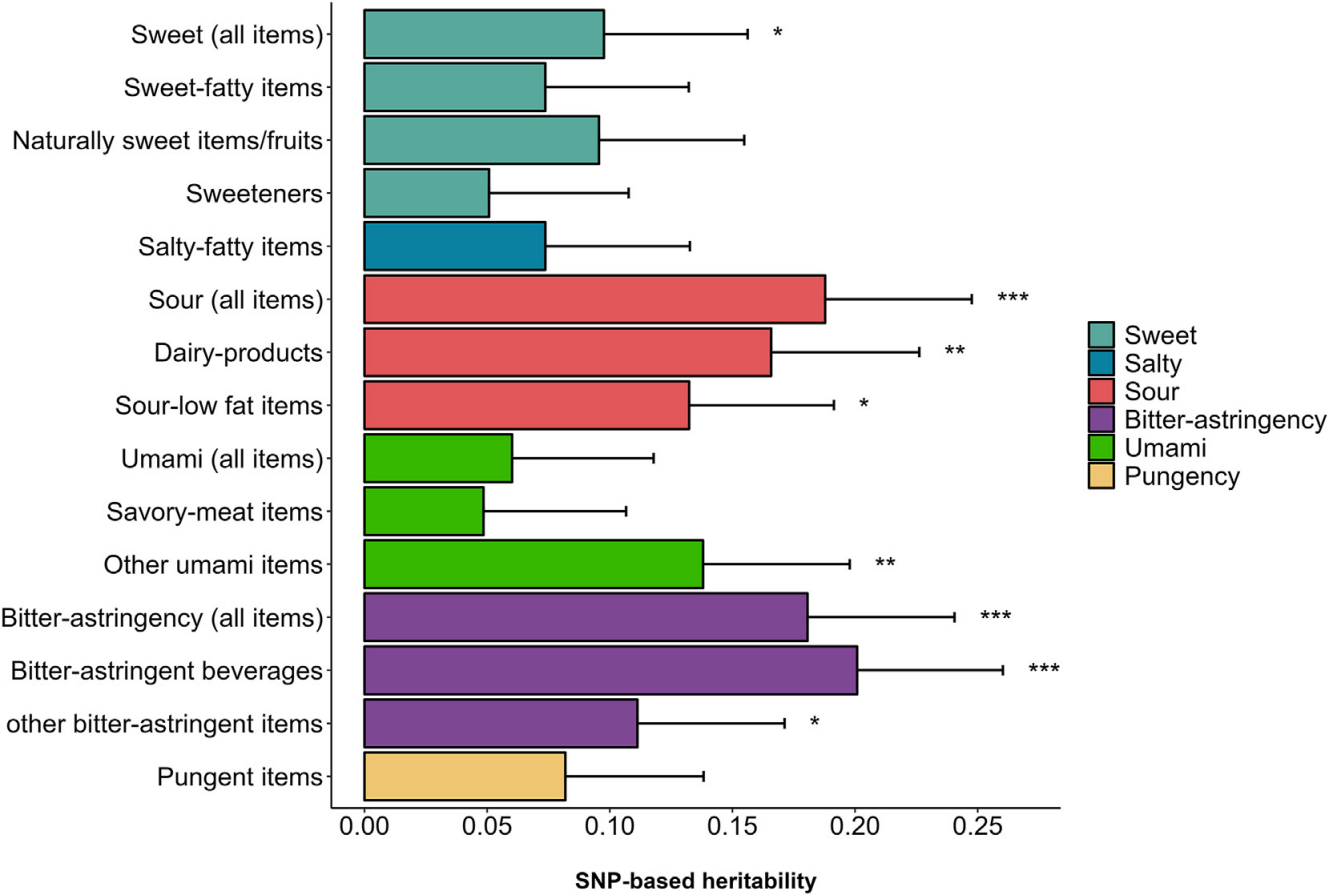
SNP-based heritability of taste liking. Estimates are shown for the 6 modalities and underlying factors of the taste liking questionnaire. Heritability was estimated with GCTA-GREML using the rank-based inverse normal transformed liking scores, and covariates (age, age^2^, sex) were included in the analysis. The error bars represent standard errors. Significance levels are denoted as ∗ *P* < 0.05, ∗∗ *P* < 0.01, and ∗∗∗ *P* < 0.001. GCTA; Genome-wide complex trait analysis; GREML, Genomic-Relatedness-Matrix Restricted Maximum Likelihood; SNP, single nucleotide polymorphism.

SNP-based heritability estimates of taste liking and underlying factors of each modality ranged between 0.05 and 0.20 when adjusting for covariates (age, age^2^, and sex) (Figure 2). The highest estimates were observed for liking of bitter-astringent beverages, sour and bitter taste modalities with h^2^ (SE) of 0.20 (0.06), 0.19 (0.06), and 0.18 (0.06) (*P* < 0.001), respectively.

### Genetic correlations

**FIGURE 3:**
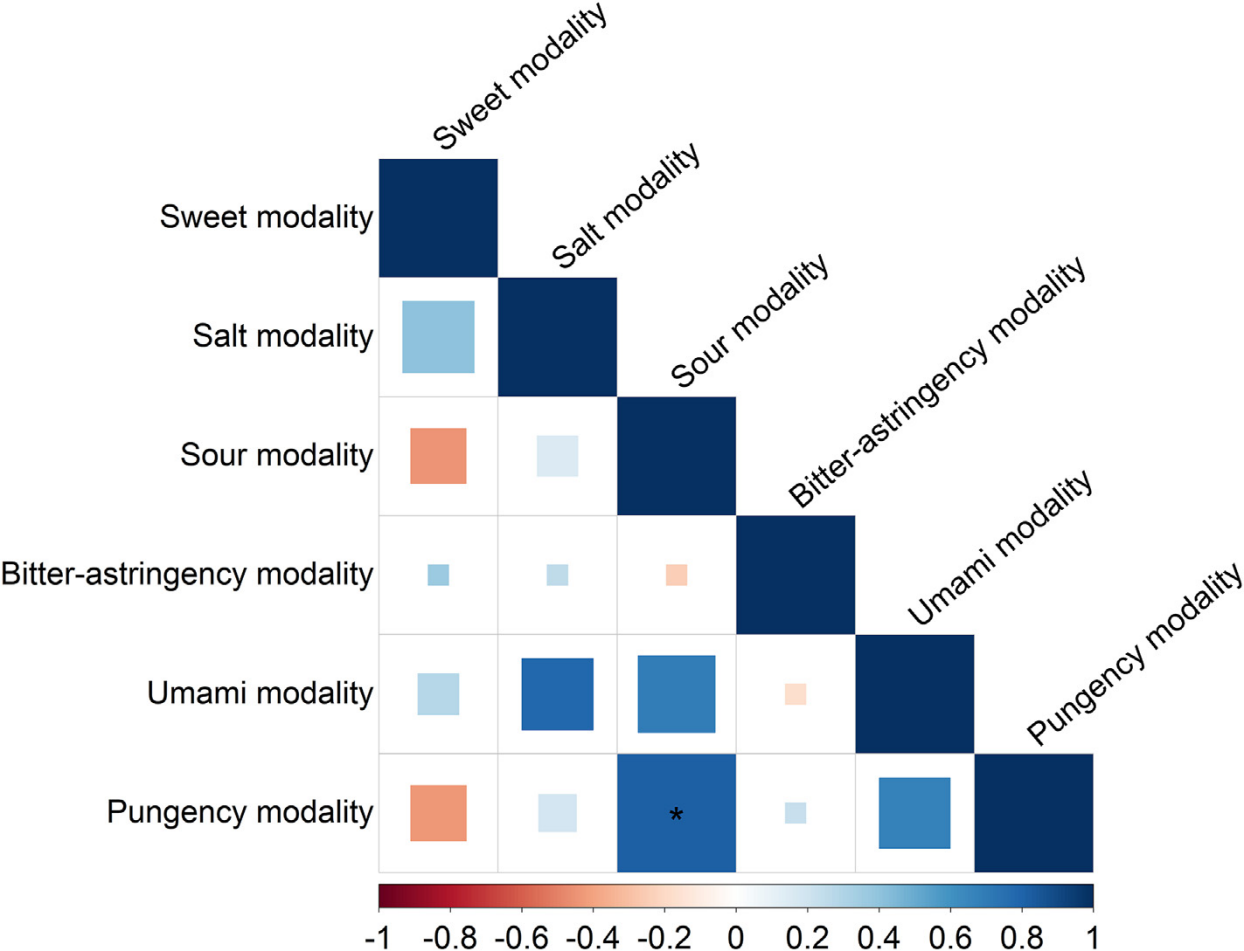
Genetic correlation among taste liking phenotypes. Bivariate genetic correlations were estimated among the 6 taste modalities of the taste liking questionnaire with linkage disequilibrium score regression. The false discovery rate was used to adjust *P* values based on 5 phenotypes. Larger squares indicate higher level of significance after adjustment. ∗Indicates an adjusted *P* < 0.01.

Among taste modalities, significant positive genetic correlation was found between sour and pungency liking (*r*_g_ = 0.81, *P*-adjusted = 0.001) (Figure 3). When analyzing the correlation with 33 phenotypes, similar findings were noted with a significant positive correlation between sour liking from TasteLQ and liking for spicy food (*r*_g_ = 0.48) and capsicum (*r*_g_ = 0.39) from UK Biobank (*P*-adjusted = 0.025) (Supplemental Figure 9). Pungency liking from TasteLQ was also significantly correlated with the latter 2 UK Biobank phenotypes (*r*_g_ = 0.63 and 0.57, *P*-adjusted = 3.1 × 10^−3^ and 3.3 × 10^−3^, respectively). However, we did not identify significant genetic correlations with the remaining phenotypes (Supplemental Figure 9).

## Discussion

In this GWAS of taste liking in the Danish adult population, we identified 1 genome-wide significant signal associated with umami liking. Additionally, when analyzing individual food items, we identified several genome-wide significant SNPs within a locus associated with liking of the bitter-tasting food item, rocket salad.

The genome-wide significant SNP, rs170518, was associated with the umami factor represented by 2 glutamate-rich food items. Functional evidence from Open Targets suggests a potential link between this SNP and *NR3C1* encoding the glucocorticoid receptor, which acts as a transcription factor, and with primary transcripts being expressed in brain and whole blood [42]. The implication of this gene in taste perception and liking may be supported by several explanations. First, animal studies indicated that the glucocorticoid receptor is expressed in mouse type 2 taste receptor cells, particularly in those sensitive to umami and sweet tastants, and in olfactory receptor neurons [43,44]. In addition, stress might modulate peripheral taste by reducing sweet and umami taste receptor expression through glucocorticoid signaling activation [43,45]. Glucocorticoids are known to influence food behaviors via the hypothalamic–pituitary–adrenal axis and directly affect food reward circuitry or act through mediators, such as insulin, neuropeptide Y, and leptin [43,46]. Remarkably, *NR3C1* variants have been linked to variations in glucocorticoid sensitivity at the cellular level, potentially influencing food preferences and palatable food intake [46,47]. Other studies in human pathology have also associated *NR3C1* methylation to mental disorders, lifestyle, and behavioral factors [48]. However, this variant might also be linked to other genes such as *HMHB1* and *YIPF5*. *HMHB1* encodes one of the minor histocompatibility antigens. These peptides play a role in immune response when complexed with the major histocompatibility complex, which has been associated with odor signaling and preferences in humans [18,49]. *YIPF5* encodes a member of the 5-pass transmembrane protein family localizing in the Golgi apparatus and the endoplasmic reticulum and is involved in vesicle-mediated transport. It should be noted that no other SNPs were in high LD with the lead SNP rs170518, which appeared isolated, requiring further explanation regarding its functional significance. Fine-mapping methods provided initial evidence supporting the credibility of this SNP and its association with umami liking, but further validation is required to confirm its causal role. To date, the only potential replication resource would be a previous GWAS of food-liking traits involving 161,625 participants from the UK Biobank [4]. However, the SNP rs170518 was not reported to be associated with soy sauce or hard cheese liking in that study.

Sensitivity analysis by adjusting the GWAS results for alcohol consumption resulted in a minor reduction of the SNP rs170518 effect on umami taste liking. This suggests that lifestyle and other environmental factors could possibly influence taste perception and liking as previously shown [50,51]. This was also supported by the low heritability estimates of taste-liking phenotypes, which supports the contribution of environmental factors. Twin and family-based studies have previously established the genetic influence on food and taste liking [52]. Our results were also in line with heritability estimates of food liking from a recent UK Biobank study [4]. Additionally, heritability in other behavioral traits like alcohol consumption is likewise comparably small [53]. Taken together, our findings indicate a plausible role of rs170518 in umami taste liking, although it is not excluded that lifestyle factors, such as alcohol consumption, smoking, and physical activity, could possibly modulate the effect.

In the GWAS of individual food items, 4 genome-wide significant variants in *TAS2R38* and *MGAM* were associated with rocket salad liking. *MGAM* encodes a brush border membrane maltase–glucoamylase protein. Concordant results were also observed when examining the association between *TAS2R38* haplotypes and taste-liking phenotypes. These findings align with previous associations of *TAS2R38* variants with bitter taste sensitivity and the liking of horseradish, grapefruit, salty food, and alcoholic beverages [4]. The identification of *TAS2R38* in association with a specific bitter vegetable in our population, although showing only weak associations with other vegetables in the UK Biobank food-liking GWAS [4], underscores the variability of taste perception and liking across populations. It also suggests that, within the same food groups, there are differences among food items, including variations in the concentration of bitter compounds, which may be influenced by several factors, such as cooking methods and processing [54,55]. Unlike other bitter vegetables, rocket salad is typically consumed raw, which preserves its bitter compounds and may explain the stronger association with *TAS2R38* observed in our study.

To further explore our results, we performed a series of post-GWAS analyses. Enrichment of genome-wide significant and suggestive loci was evident in pathways related to the neuronal system, sensory perception, and signal transduction, as well as in brain regions known for their involvement in eating behavior, reward, and hedonic evaluation of tastes. These findings align with prior studies highlighting the neural basis of food liking, nutrient preferences, and behavioral traits [4,56,57].

In this study, we identified a positive genetic correlation between sour and pungency liking, as measured by the TasteLQ. This correlation with the TasteLQ sour modality was comparable with the findings using capsicum- or spicy food-liking data from the UK Biobank, suggesting a pleiotropic effect of the genetic factors influencing the liking of both sour and pungent tastes. This overlap in genetic factors may be explained by both modalities sharing common transduction pathways, primarily through the transient receptor potential channels, which detect both acidic and spicy stimuli [58,59]. Additionally, a previous twin study identified a subgroup of adventurous individuals who were more likely to enjoy both sour and spicy flavors. This subgroup was linked to *TAS1R1* and *PKD1L3* genes, further supporting the role of genetic factors [60]. However, we cannot rule out the possibility that nongenetic factors might also modulate liking of both sour and pungency, as their phenotypic correlation (Pearson, *r* = 0.43) is lower than the genetic correlation. This lower phenotypic correlation could also be attributed to measurement errors from the self-reported liking in the TasteLQ [4].

The limited detection of genome-wide significant loci in the present study may be partly explained by the polygenic nature of the measured taste-liking phenotypes and the observed pleiotropic effects, where genetic variants influencing taste liking may also affect metabolic or behavioral phenotypes. This was shown in the phenome-wide association study analysis, where the umami-lead SNP was associated with nutritional, psychiatric, and metabolic traits, although significance was lost after adjusting for multiple comparisons. Among the nutritional traits, we note the oily fish intake, which can be classified as an umami-taste item [61]. Although this may suggest a potential involvement of the umami-lead SNP in dietary intake, the association did not survive correction for multiple testing. We cannot exclude that our small sample size limited the statistical power to capture signals with moderate or small effects. In this context, we expected that SNPs associated with taste perception, food liking, and consumption in previous studies would be at least nominally associated in our data. Indeed, in the taste-related SNP analysis, associations were confirmed for many of these loci with relatively low effect sizes, for instance, *FTO*, *FGF21*, and *ADH1B*. Of note, discrepancies exist in the methods used to assess liking or preferences across previous studies, as well as in phenotype definitions, which can differ from our measured phenotype and may explain the variation in the observed associations in our taste-related SNP analysis.

We cannot exclude the possibility that the high number of GWASs tested in this study along with the limited power due to the relatively low sample size might introduce a high risk for type I errors. Additionally, we did not apply a study-wide significance threshold to account for the number of the GWASs. Although we found biologically plausible explanations for the association of the rs170518 locus with taste liking and its potential role supported by fine-mapping analyses, this locus remains a preliminary finding that requires further replication in an independent population. Despite the strength of this study in using a newly developed and valid TasteLQ, recalled liking scores may be influenced by memory and personal experiences, introducing potential self-report bias. This bias may be also more relevant in older populations, as our studied population presented a relatively high median age. Additionally, taste preferences vary across different life stages, and an inverse relationship between age and taste intensity has been previously reported [17,62]. Therefore, we can expect physiological changes in our population that may affect taste acuity as well as liking ratings, which in turn could impact the genotype–phenotype relationship. Thus, we cannot generalize our results to other age groups. Although several covariates were considered in the GWAS analysis, including age and lifestyle factors, additional environmental factors might be involved and could be interesting to explore in future studies. As this study focused on the Danish population, generalizing our findings to populations of different ancestries requires further validation. Finally, a larger sample size could possibly validate true signals among the identified suggestive loci.

In conclusion, we found loci implicated in taste liking that are preliminary and require further confirmation and follow-up to better understand the biological mechanism underlying the relationship between liking and dietary consumption. Our results also supported some of the previous findings highlighting the complexity and heterogeneity of dietary behavioral traits, while providing additional insights into the existing data. Knowledge of genomic variation in taste liking enables targeted nutrition strategies, thereby potentially paving an avenue with dietary public appeals for prevention of cardiometabolic disorders.

## Author contributions

The authors’ responsibilities were as follows – SH, CCK, OP, WB, TH, NG: involved in the conceptualization; SH, CCK: responsible for the methodology, formal analysis, data interpretation, and writing the first draft of the manuscript; AL, LLK, NG: provided and supervised access to data from the health examination studies; NG: provided fund acquisition and supervised the study; and all authors: read and approved the final manuscript.

## Data availability

The data generated and analyzed during this study are included in this article and its supplementary material. Restrictions apply to the availability of data related to cohorts according to Danish legislation. Therefore, the data and the information regarding the participants cannot be publicly available. A request for access to the data or collaboration can be sent to the authors AL, LLK, TH, and NG.

## Ethical approval

This study was covered by the approval of the Ethical Committee [Inter99 (KA 98 155), Health2006 and Health2008 (KA-20060011), Health2010 (H-4-2009-124), and DanFunD (H-3-2011-081 and H-3-2012-0015)] and the Danish Data Protection agency (journal number P-2020-1074). All participants provided an informed consent.

## Funding

This work was supported by the Novo Nordisk Foundation (grant numbers: NNF18CC0034900 and NNF23SA0084103). CCK was supported by the Danish Diabetes and Endocrine Academy, which is funded by the Novo Nordisk Foundation (grant ID: PhD0040-20).

## Conflict of interest

The authors declare that they have no known competing financial interests or personal relationships that could have appeared to influence the work reported in this article. NG and CCK are currently employed at Novo Nordisk.
