# Supplementary material for: A Genome-Wide Association Study of Taste Liking in the Danish Population
Source: J Nutr. 2025 Jun 7;155(8):2591–601. doi: 10.1016/j.tjnut.2025.06.001 (PMC12405901; doi:10.1016/j.tjnut.2025.06.001)
Supplement: multimedia component 1 [file mmc1.pdf]

Supplementary Material

A genome-wide association study of taste liking in the Danish population

1st authors: Sara Haydar & Camilla Cederbye Karlsson

Contents

Supplementary Results

Supplementary Figures

Supplementary Figure 1. Overview of taste-liking phenotypes used in the GWAS analysis. 4

Supplementary Figure 2. Quantile-quantile plots of P-values from taste liking genome-wide association studies. 5

Supplementary Figure 3. Distribution of liking scores of other umami-items factor by genotypes of the genome-wide significant SNP rs170518 in the study population (n=6,347). 6

Supplementary Figure 4. LD plot and haplotypes within the region of the lead SNP rs170518 in the study population. 7

Supplementary Figure 5. Manhattan plots of the taste liking GWAS results (n=6,347). 8

Supplementary Figure 6. Liking scores across *TAS2R38* phenotypes in the Danish population. 9

Supplementary Figure 7. Tissue expression analysis results for taste liking. 10

Supplementary Figure 8. Phenome-wide association study. 11

Supplementary Figure 9. Genetic correlation between taste liking and 33 phenotypes. 12

Supplementary Tables

Supplementary Table 1. Description of Danish cohorts from population-based studies included in the analysis 13

Supplementary Table 2. List of 28 SNPs previously reported with taste perception, food liking and intake 14

Supplementary Table 3. List of the 33 phenotypes included in the genetic correlations analysis 16

Supplementary Table 4. Association of haplotypes pairs within the rs170518 region with other-umami items factor liking 17

Supplementary Table 5. Association results of previously reported taste-related SNPs with taste-liking phenotypes in the study population 18

Supplementary Table 6. Pathway enrichment analysis in GeneNetwork 21

Supplementary References

Supplementary Results

Haplotype analysis of *TAS2R38*

The estimation of *TAS2R38* haplotypes using the 3 SNPs (rs10246939, rs1726866, rs713598) revealed 2 major haplotypes, AVI and PAV, with frequencies of 58% and 38%, respectively. Two haplotypes were significantly associated with liking of other-astringent bitter items. The PAV haplotype was negatively associated with $P<8x10^{-3}$, while the AVI haplotype was positively associated with $P<5x10^{-3}$ (data not shown). When considering the haplotype pairs, a total of 15% of the population were carriers of PAV/PAV which confers the *super-taster* phenotype. The *taster* phenotype was represented by PAV/AVI (44%) and PAV/AAV (3%), while 33 % of the population were carriers of the AVI/AVI corresponding to the *non-taster* phenotype. Additionally, an *intermediate* taster phenotype (5%) was found with 1 AVI copy corresponding to AAV/AVI. To compare the liking scores across the haplotype pairs, we followed the above phenotype classification (*super-taster, taster, intermediate, non-taster*) and as previously described (1), while we removed 23 individuals from the analysis due to the low frequency of the genotypes. No significant differences were observed in liking scores of the 6 modalities across the different phenotypes (Supplementary Figure S6). However, within the underlying factors, a significant difference was detected for the other-astringent bitter items between the *super-tasters* and *non-tasters* (P-adjusted=$7.8x10^{-3}$). Results were similar to those obtained for rocket salad liking where a significant difference was observed between *super-tasters and the other phenotypes* (lowest adjusted P-value being between *super-tasters and non-tasters*; P= 2.10×10^{-9}) (Supplementary Figure S6).

Supplementary Figures

Modality	Underlying factors per modality	Items
**Sweet**	*Sweet-fatty items*	Cookies, icecream, cream puffs, brownie, chocolate spread, milk chocolate, Danish pastry
	Naturally sweet items/fruits	Ripe honeydew melon, ripe pear, ripe strawberry
	Sweeteners	Honey, syrup
**Salty**	*Salty-fatty items*	Chips, salted popcorn, salted peanuts, salted crackers, French fries
**Sour**	*Dairy products*	Natural yoghurt, creme fraiche, buttermilk
	Sour-low fat items	Vinegar, lemon, red currant, pickles, unripe green apple
**Bitter-astringency**	*Bitter-astringent beverages*	Red wine, beer, tonic water, coffee
	Other bitter-astringent items	Rocket salad, walnuts, dark chocolate, black tea
**Umami**	*Savory-meat items*	Bacon, salami, beef steak, chicken soup
	Other umami items	Soy sauce, parmesan cheese
**Pungency**	*Pungent items*	Black pepper, chili, Dijon mustard, ginger, raw onion

Supplementary Figure 1. Overview of taste-liking phenotypes used in the GWAS analysis. Food items and underlying factors representing each modality from the TasteLQ. The salty and pungency modalities consist of 1 underlying factor (2).

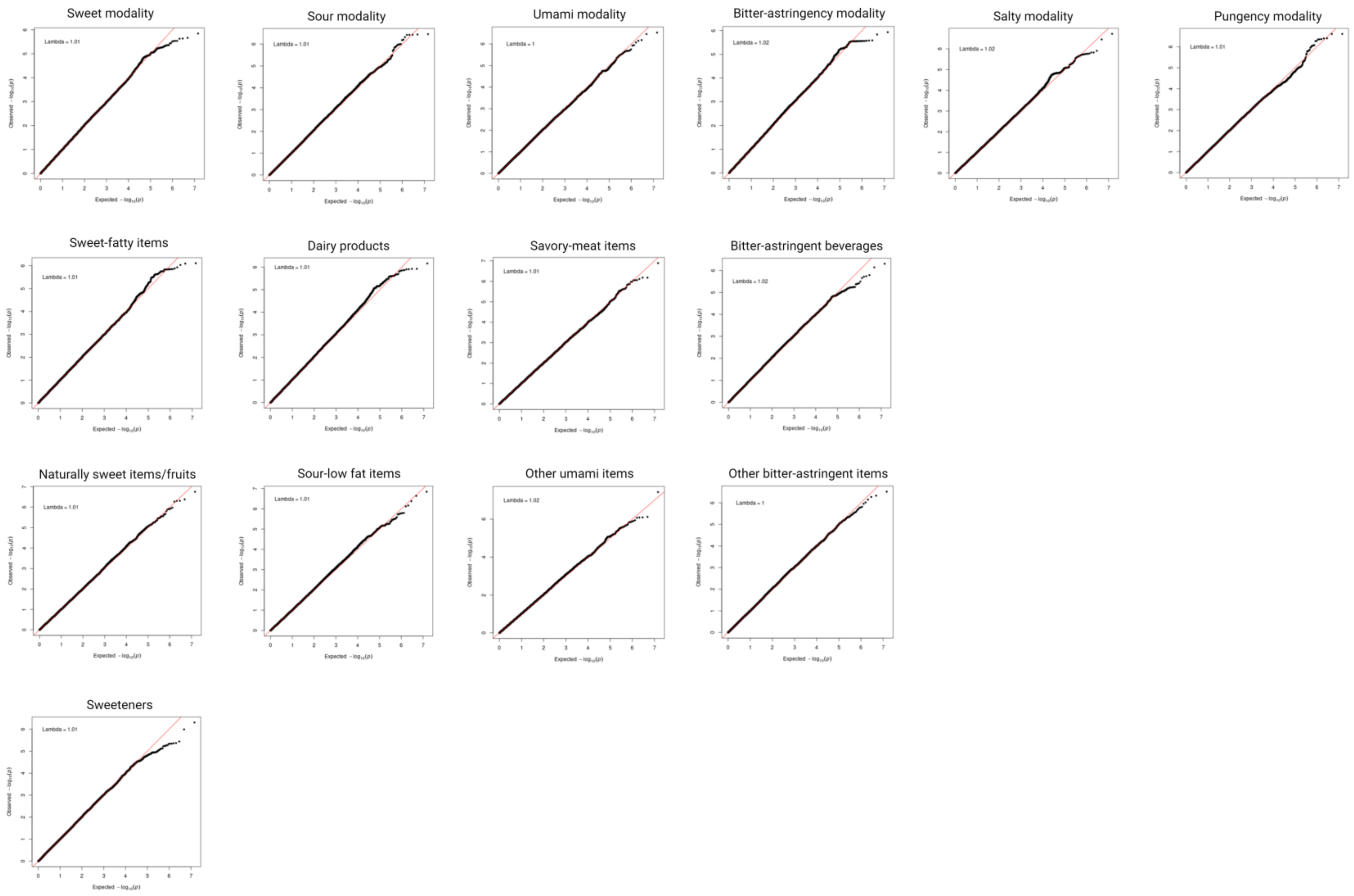

Supplementary Figure 2. Quantile-quantile plots of P-values from taste liking genome-wide association studies.

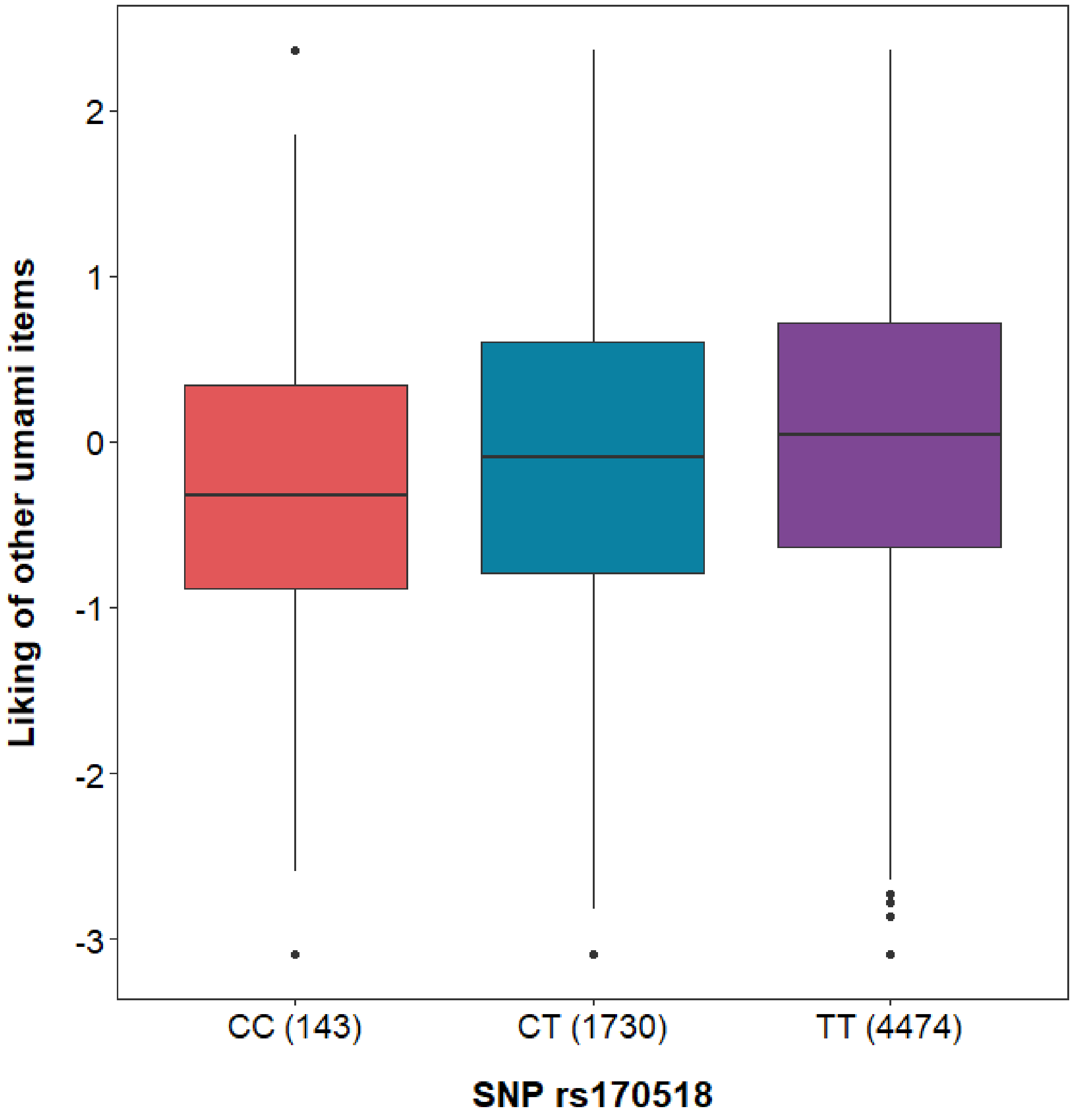

Supplementary Figure 3. Distribution of liking scores of other umami-items factor by genotypes of the genome-wide significant SNP rs170518 in the study population (n=6,347). Results are displayed as rank-based inverse normal transformed liking scores.

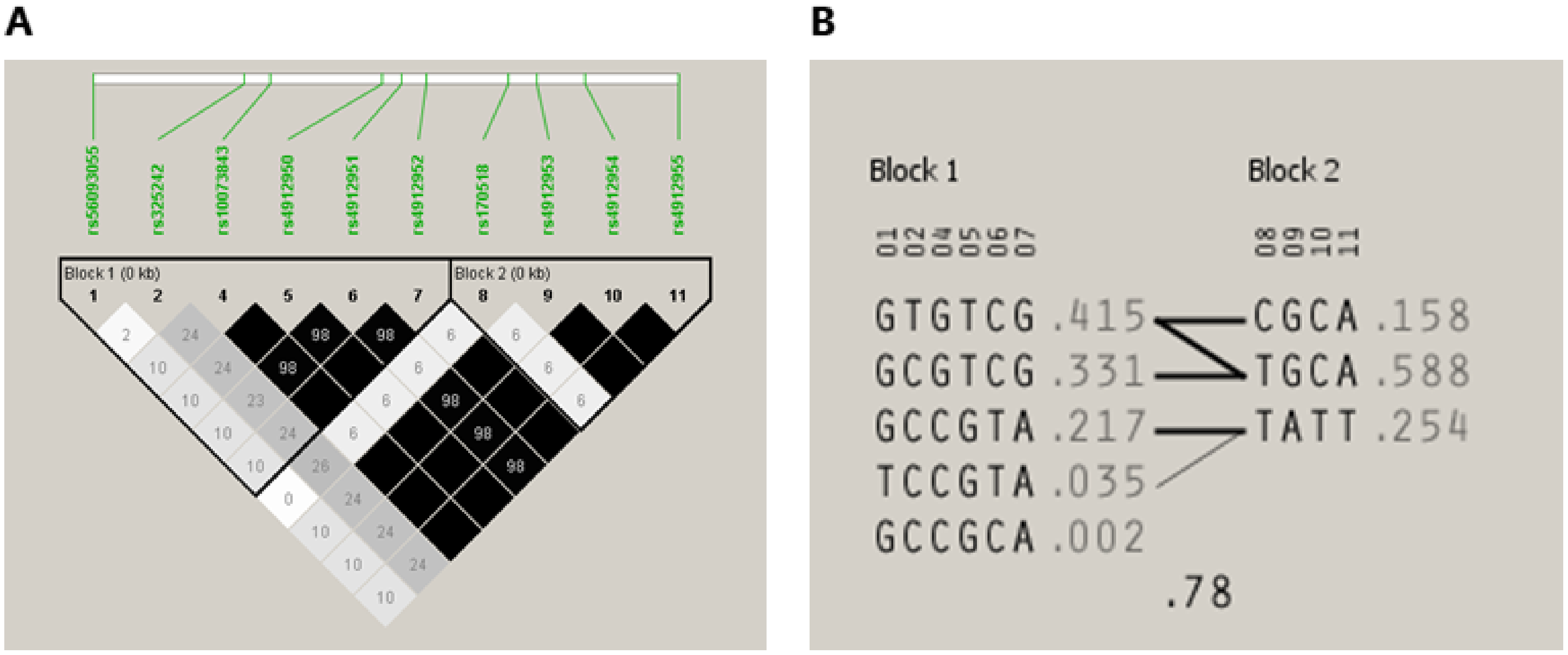

Supplementary Figure 4. LD plot and haplotypes within the region of the lead SNP rs170518 in the study population. Block 1 comprising 6 SNPs, and block 2 included the genome-wide significant variant rs170518 associated with other umami items factor liking. LD blocks and haplotypes frequencies were visualized in HAPLOVIEW software using an unrelated sample from the study population as input (n=5,964). A) Plot showing R2 values for pairwise LD, B) Haplotype population frequencies in block 1 and 2: LD, Linkage disequilibrium.

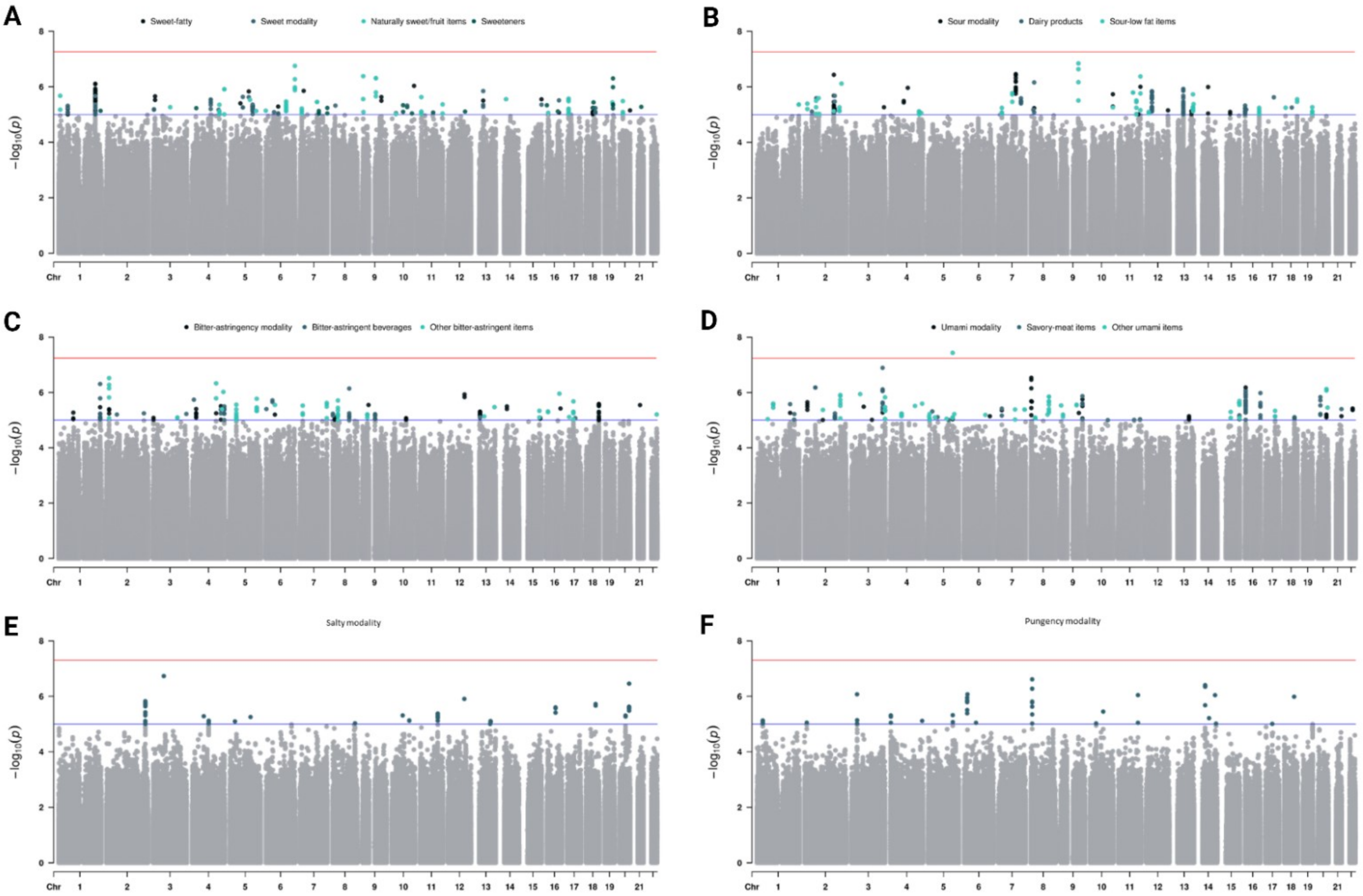

Supplementary Figure 5. Manhattan plots of the taste liking GWAS results (n=6,347). A) Sweet, B) Sour, C) Bitter-astringency, D) Umami, E) Salt, F) Pungency. The blue horizontal line represents the genome-wide suggestive threshold ($P<1x10^{-5}$) and the red line represents the genome-wide significant threshold ($P<5x10^{-8}$). The different dot colors in each panel highlights SNPs associated by modality and underlying factors. Results represent the basic model using the rank-based inverse normal transformed liking scores and adjusted for age, age^2, sex. Genomic locations are shown as GRCh37/hg19.

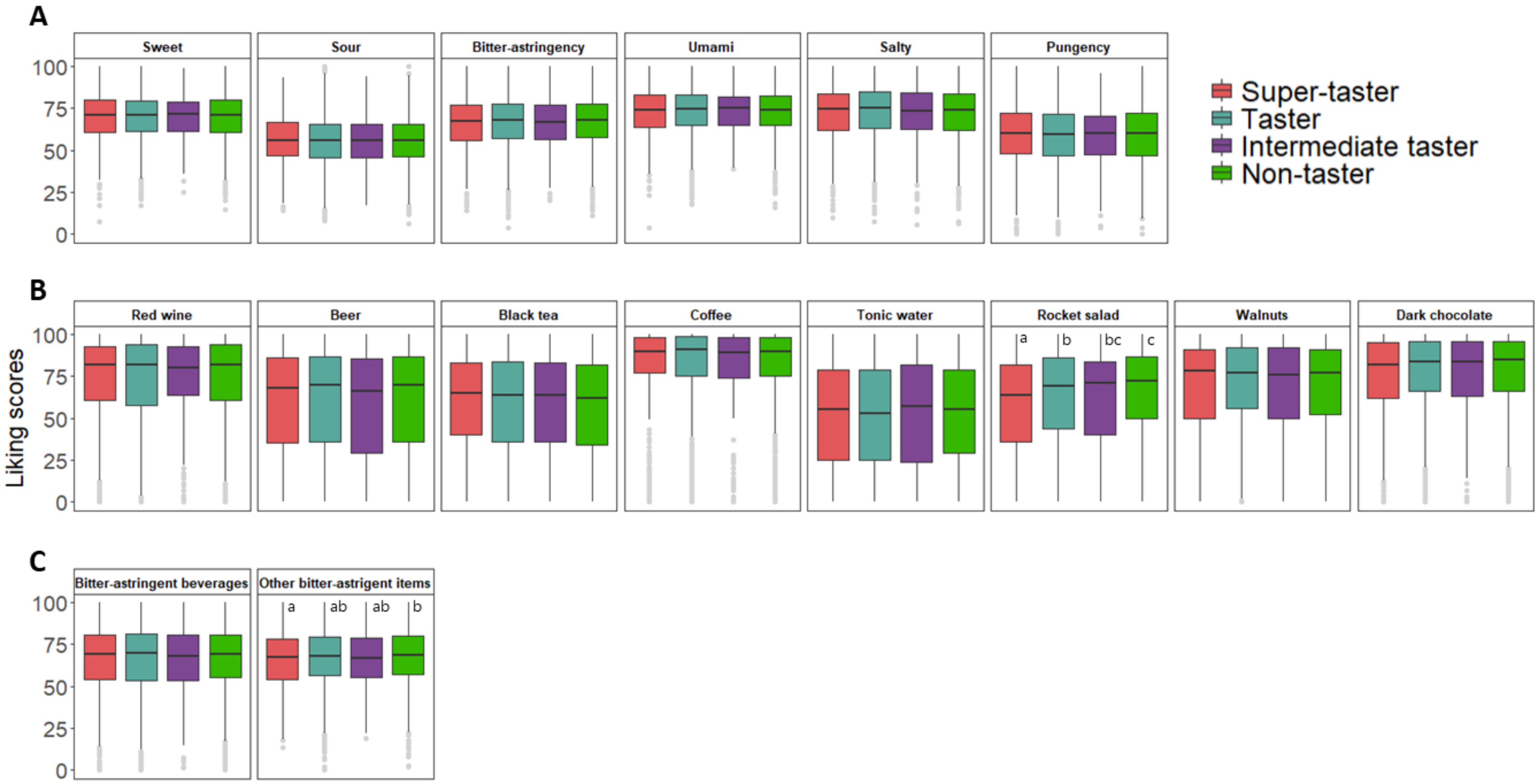

Supplementary Figure 6. Liking scores across *TAS2R38* phenotypes in the Danish population. Scores are presented for A) modalities, B) bitter-astringent food items, C) factors underlying the bitter-astringency modality. Haplotypes were determined using the three variants (rs10246939, rs1726866, rs713598), and assigned to individuals as haplotype pairs with PHASE program. Super-taster consists of PAV/PAV; Taster: PAV/AVI and PAV/AAV; Intermediate taster: AAV/AVI; Non-taster: AVI/AVI. Data are presented as median and IQR. Letters are only shown when statistical differences are detected using Wilcoxon rank sum test with Bonferroni adjustment ($P<0.05$). Groups with different letters are significantly different from each other; IQR, Interquartile range.

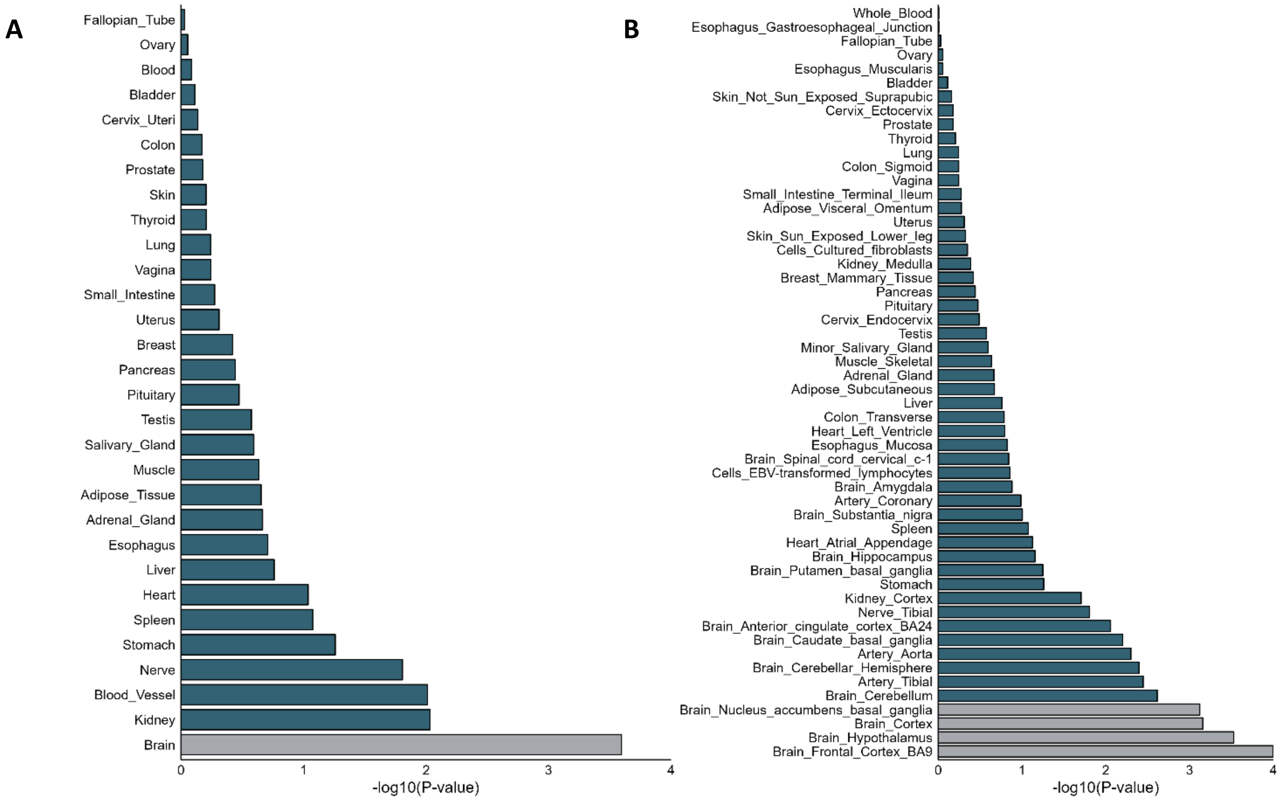

Supplementary Figure 7. Tissue expression analysis results for taste liking. Analysis was done in FUMA (GTEx v8) using genome-wide and suggestively associated genes with the 6 modalities and underlying factors of the TasteLQ. Results are ordered by two-side DEG P-value. Grey bars indicate significant enrichment with $P_{Bonferroni}$<0.05. A) 30 general tissue types, B) 54 specific tissue types; DEG, differentially expressed genes; FUMA, Functional mapping and annotation of GWAS.

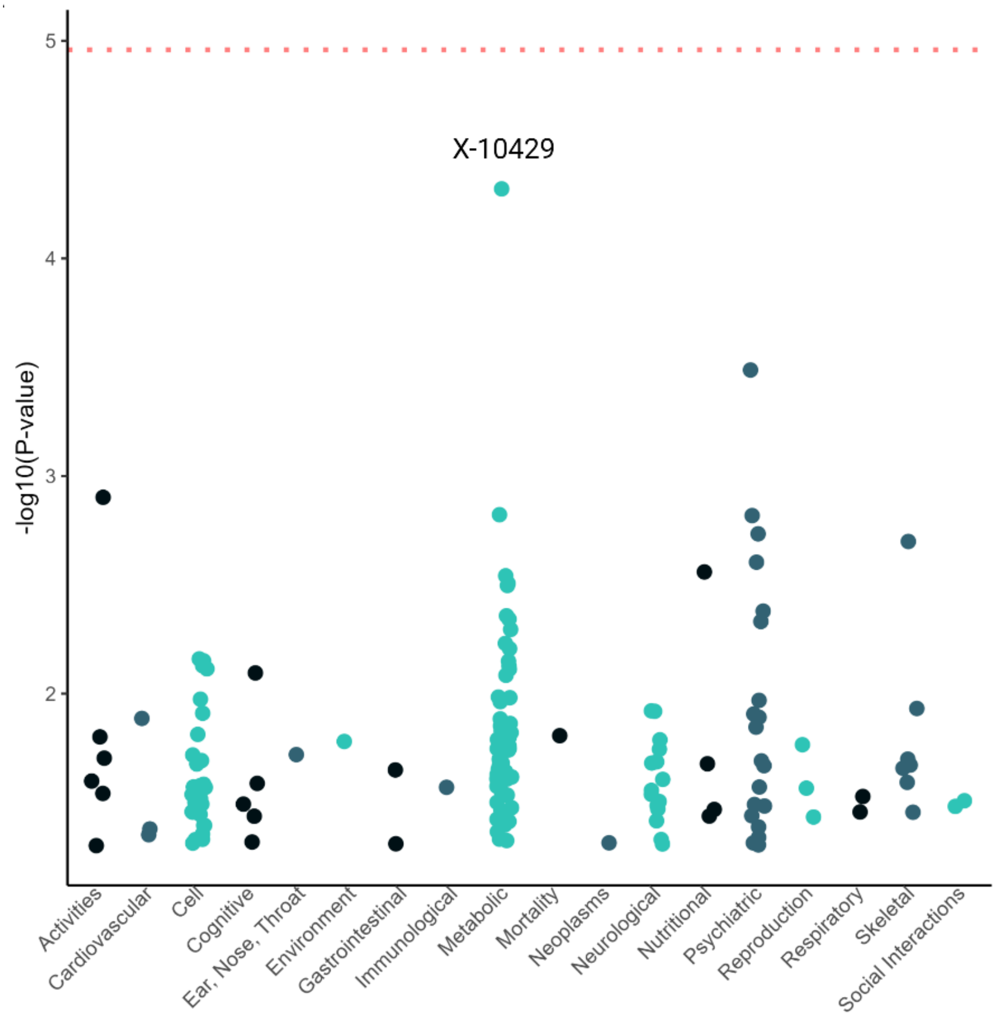

Supplementary Figure 8. Phenome-wide association study. Associations between the genome-wide significant variant rs170518 and different traits across different domains using the GWAS Atlas online tool. The red line corresponds to Bonferroni correction threshold (P=0.05/4,756=1.1x10^{-5}); X-10429, unknown metabolite.

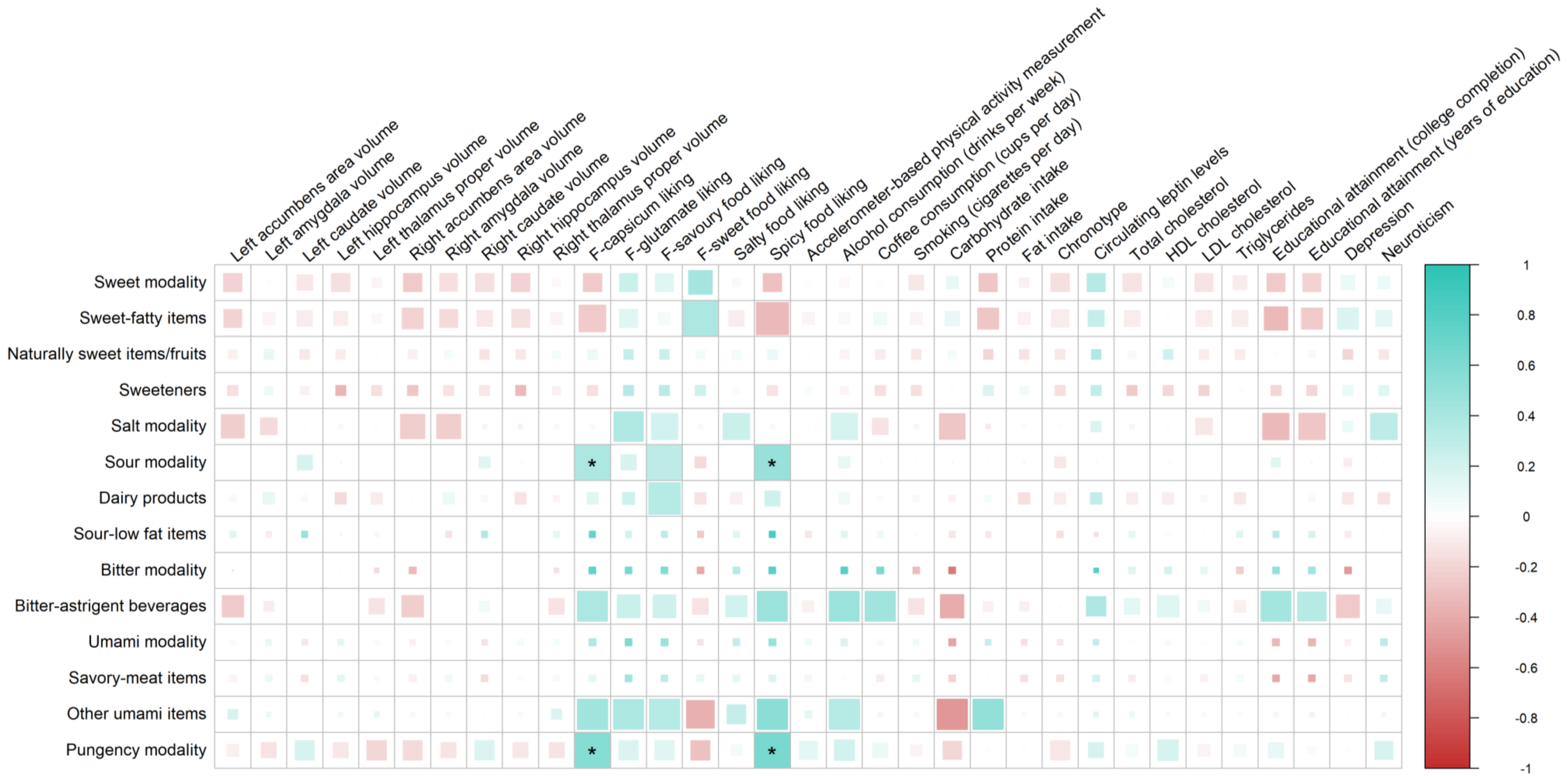

Supplementary Figure 9. Genetic correlation between taste liking and 33 phenotypes. Bivariate genetic correlations were estimated using LD-score regression with LDSC. Green color indicates positive correlation, while the red indicates negative correlation. The other bitter-astringent items factor from the taste-liking phenotypes was excluded from analysis due to error related to a low sample size. The false discovery rate was used to adjust P-values based on 33 phenotypes. Larger squares indicate higher level of significance after adjustment. *indicates an adjusted P<0.05; LD, Linkage disequilibrium.

Supplementary Tables

Supplementary Table 1. Description of Danish cohorts from population-based studies included in the analysis

Cohort	Description	Sample size*	Reference
**Inter99**	Inter99 is a Danish intervention study that was conducted in the period 1999-2006 including randomly selected individuals in the ages 30-60 years from the south-western part of Copenhagen County. Individuals were invited to a health screening program, a personal risk assessment, and lifestyle counseling.	1,830	Jørgensen et al., 2003; PMID: 14663300
**DanFunD**	The Danish study for Functional Diseases is a population-based study conducted in the period 2011-2015 including random sample with individuals aged 18-76 years and living in the western part of the greater Copenhagen. The aim of this study was to determine the epidemiology of functional somatic syndromes.	2,780	Dantoft et al., 2017; PMID: 28275316
**Health2006**	Health2006 included randomly selected individuals aged 18-69 years from the Danish population and living in the south-western part of the greater Copenhagen area. The study was conducted in the period 2006-2008. Participants went through a thorough health examination in which blood pressure, blood samples and questionnaire data were obtained.	983	Thuesen et al., 2014; PMID: 23615486
**Health2008**	Health2008 was conducted in the period 2008-2009 and included randomly selected individuals between 30-60 years and living in the western part of Copenhagen. At the study examination, different factors like blood pressure and cholesterol were assessed and questionnaire data on diet and socioeconomic status amongst others, were collected.	277	Byberg et al., 2012; PMID: 22587629
**Health2010**	Health2010 study was conducted between 2010-2011 and included a random sample aged 18-69 years and living in the western part of the capital region of Denmark. The study focused on cardiovascular disease, diabetes, asthma, and allergy. Different measures were recorded during examination including blood pressure, blood samples, and dietary data obtained via questionnaire.	477	Aadahl et al., 2014; PMID: 25113139

The age range represents the age at initial recruitment. * Sample size from each cohort included in this study.

Supplementary Table 2. List of 28 SNPs previously reported with taste perception, food liking and intake

rsID	Gene	Chr	Position	Reported phenotype	Reference
rs10889761	*COX6B1P7, ELOCP18**	1	68764949	Perceived intensity of sucrose	Hwang et al., 2019; PMID:31005972
rs35744813	*TAS1R3*	1	1265460	Suprathreshold sensitivity to sucrose<br>Sucrose sensitivity scores	Chamoun et al., 2021; PMID:33444969<br>Fushan et al., 2009; PMID:19559618
rs1260326	*GCKR*	2	27730940	Caffeine consumption from coffee or tea<br>Bitter alcoholic beverage consumption<br>Bitter beverage consumption	Said et al., 2020; PMID:33287642<br>Zhong et al., 2019; PMID:31046077<br>Zhong et al., 2019; PMID:31046077
rs504675	*LINC01833*	2	45154689	F-bitter food liking (derived food-liking factor)<br>F-burn/spicy liking (derived food-liking factor)	May-Wilson et al., 2022; PMID:35585065<br>May-Wilson et al., 2022; PMID:35585065
rs2733520	*TBC1D5*	3	17246392	F-fatty/dairy food liking (derived food-liking factor)	May-Wilson et al., 2022; PMID:35585065
rs2733481	*TBC1D5*	3	17320397	F-meat liking (derived food-liking factor)	May-Wilson et al., 2022; PMID:35585065
rs7619139	*RARB*	3	25110415	Raw vegetable consumption<br>F-salad vegetables liking (derived food-liking factor)<br>Cabbage liking	Cole et al., 2020; PMID:32193382<br>May-Wilson et al., 2022; PMID:35585065<br>May-Wilson et al., 2022; PMID:35585065
rs7429279	*RARB*	3	25118637	F-sweet food liking (derived food-liking factor)<br>F-cake/biscuits liking (derived food-liking factor)	May-Wilson et al., 2022; PMID:35585065<br>May-Wilson et al., 2022; PMID:35585065
rs62250717	*CADM2*	3	85521990	Beef/steak liking	May-Wilson et al., 2022; PMID:35585065
rs12629607	*CADM2*	3	85583608	F-savoury food liking (derived food-liking factor)	May-Wilson et al., 2022; PMID:35585065
rs7652808	*CADM2*	3	85603643	F-fatty/dairy food liking (derived food-liking factor)	May-Wilson et al., 2022; PMID:35585065
rs2029130	*CADM2*	3	85644047	Spicy food liking	May-Wilson et al., 2022; PMID:35585065
rs1229984	*ADH1B*	4	100239319	F-coffee/alcohol liking (derived food-liking factor)<br>Bitter alcoholic beverage consumption	May-Wilson et al., 2022; PMID:35585065<br>Zhong et al., 2019; PMID:31046077
rs710360	*LINC02144, TMEM161B**	5	87144026	F-dairy liking (derived food-liking factor)	May-Wilson et al., 2022; PMID:35585065
rs9257566	*OR2J2, OR2J4P**	6	29144532	Horseradish liking	May-Wilson et al., 2022; PMID:35585065
rs1726866	*MGAM, TAS2R38*	7	141672705	Bitter taste perception (6-n-propylthiouracil) in obesity with metabolic syndrome<br>Grapefruit liking	Coltell et al., 2019; PMID:31005965<br>May-Wilson et al., 2022; PMID:35585065
rs2409764	*FAM167A-AS1, FAM167A*	8	11281273	F-fatty/dairy food liking (derived food-liking factor)	May-Wilson et al., 2022; PMID:35585065
rs2472297	*CYP1A2, CYP1A1*	15	75027880	Bitter non-alcoholic beverage consumption<br>Bitter beverage consumption<br>Caffeine consumption from coffee	Zhong et al., 2019; PMID:31046077<br>Zhong et al., 2019; PMID:31046077<br>Said et al., 2020; PMID:33287642
rs11642015	*FTO*	16	53802494	Salami liking	May-Wilson et al., 2022; PMID:35585065
rs10852521	*FTO*	16	53804965	Honey liking	May-Wilson et al., 2022; PMID:35585065
rs55872725	*FTO*	16	53809123	F-fatty/salty food liking (derived food-liking factor)	May-Wilson et al., 2022; PMID:35585065
rs72805612	*FTO*	16	53834608	Crisps liking	May-Wilson et al., 2022; PMID:35585065
rs9929436	*CDH13*	16	83661215	Salami liking	May-Wilson et al., 2022; PMID:35585065

rs11859365	*CDH13*	16	83683945	F-sharp flavour liking (derived food-liking factor)	May-Wilson et al., 2022;	PMID:35585065
rs224537	*TRPV1*	17	3487202	Spicy food liking	May-Wilson et al., 2022;	PMID:35585065
rs4939727	*DCC*	18	50792914	F-capsicum liking (derived food-liking factor)	May-Wilson et al., 2022;	PMID:35585065
rs7256200	*APOC1*	19	45415935	F-glutamate liking (derived food-liking factor)	May-Wilson et al., 2022;	PMID:35585065
rs838133	*FGF21*	19	49259529	Dietary macronutrient intake (protein)	Chu et al., 2013;	PMID:23372041
				Dietary macronutrient intake (carbohydrates)	Merino et al., 2018;	PMID:29988085
				Relative carbohydrate intake	Merino et al., 2022;	PMID:34426670
				Relative protein intake	Merino et al., 2022;	PMID:34426670
				F-cake/biscuits liking (derived food-liking factor)	May-Wilson et al., 2022;	PMID:35585065
				F-glutamate liking (derived food-liking factor)	May-Wilson et al., 2022;	PMID:35585065
				F-savoury food liking (derived food-liking factor)	May-Wilson et al., 2022;	PMID:35585065

* Nearest upstream and downstream genes.

Supplementary Table 3. List of the 33 phenotypes included in the genetic correlations analysis

Phenotype	Reference
Left accumbens area volume	Fürtjes AE et al., 2023; PMID: 36987996
Left amygdala volume	Fürtjes AE et al., 2023; PMID: 36987996
Left caudate volume	Fürtjes AE et al., 2023; PMID: 36987996
Left hippocampus volume	Fürtjes AE et al., 2023; PMID: 36987996
Left thalamus proper volume	Fürtjes AE et al., 2023; PMID: 36987996
Right accumbens area volume	Fürtjes AE et al., 2023; PMID: 36987996
Right amygdala volume	Fürtjes AE et al., 2023; PMID: 36987996
Right caudate volume	Fürtjes AE et al., 2023; PMID: 36987996
Right hippocampus volume	Fürtjes AE et al., 2023; PMID: 36987996
Right thalamus proper volume	Fürtjes AE et al., 2023; PMID: 36987996
F-capsicum liking	May-Wilson S et al., 2022; PMID: 35585065
F-glutamate liking	May-Wilson S et al., 2022; PMID: 35585065
F-savoury food liking	May-Wilson S et al., 2022; PMID: 35585065
F-sweet food liking	May-Wilson S et al., 2022; PMID: 35585065
Salty food liking	May-Wilson S et al., 2022; PMID: 35585065
Spicy food liking	May-Wilson S et al., 2022; PMID: 35585065
Accelerometer-based physical activity measurement	Klimentidis YC et al., 2018; PMID: 29899525
Alcohol consumption (drinks per week)	Liu M et al., 2019; PMID: 30643251
Coffee consumption (cups per day)	Cole JB et al., 2020; PMID: 32193382
Smoking (cigarettes per day)	Liu M et al., 2019; PMID: 30643251
Carbohydrate intake	Merino J et al., 2022; PMID: 34426670
Protein intake	Merino J et al., 2022; PMID: 34426670
Fat intake	Merino J et al., 2022; PMID: 34426670
Chronotype	Jones SE et al., 2019; PMID: 30696823
Circulating leptin levels	Folkersen L et al., 2020; PMID: 33067605
Total cholesterol	Willer CJ et al., 2013; PMID: 24097068
HDL cholesterol	Willer CJ et al., 2013; PMID: 24097068
LDL cholesterol	Willer CJ et al., 2013; PMID: 24097068
Triglycerides	Willer CJ et al., 2013; PMID: 24097068
Educational attainment (college completion)	Rietveld CA et al., 2013; PMID: 23722424
Educational attainment (years of education)	Rietveld CA et al., 2013; PMID: 23722424
Depression	Nagel M et al., 2018; PMID: 29942085
Neuroticism	Okbay A et al., 2016; PMID: 27089181

Supplementary Table 4. Association of haplotypes pairs within the rs170518 region with other-umami items factor liking

	Genotype	Sequence	Frequency (%)	Estimate (95% CI)
**Block 1**	H1/H1	GTGTCG/GTGTCG	17.2	Reference§
	H1/H3	GTGTCG/GCGTCG	27.7	**0.090 (0.013,0.168)***
	H1/H5	GTGTCG/GCCGTA	17.7	**0.126 (0.040,0.212)****
	H1/H6	GTGTCG/TCCGTA	3	**0.220 (0.062,0.377)****
	H3/H3	GCGTCG/GCGTCG	11	0.093 (-0.005,0.191)
	H3/H5	GCGTCG/GCCGTA	13.9	**0.163 (0.071,0.254)*****
	H3/H6	GCGTCG/TCCGTA	2.2	**0.240 (0.060,0.420)****
	H5/H5	GCCGTA/GCCGTA	5.1	**0.140 (0.012,0.267)***
**Block 2**	H1/H1	**T**GCA/**T**GCA	34.5	Reference§
	H1/H2	**T**GCA/**T**ATT	29.5	**0.071 (0.008,0.135)***
	H1/H3	**T**GCA/**C**GCA	19.2	**-0.107 (-0.179,-0.035)****
	H2/H2	**T**ATT/**T**ATT	6.8	0.042 (-0.065,0.148)
	H2/H3	**T**ATT/**C**GCA	7.7	-0.063 (-0.164,0.037)
	H3/H3	**C**GCA/**C**GCA	2.3	**-0.305 (-0.479,-0.132)*****

The lead SNP rs170518 is presented in bold in the corresponding haplotype in block 2. Haplotypes were reconstructed using the PHASE program. Results are based on rank-based inverse normal transformed liking scores, adjusted for age, age^2 and sex. §Haplotype pairs used as reference in the linear regression model. *P<0.05; **P<0.01; ***P<0.001; CI, confidence interval.

Supplementary Table 5. Association results of previously reported taste-related SNPs with taste-liking phenotypes in the study population

Sweet modality

rsID	*Gene*	*Chr*	*Position*	*Tested phenotype*	*Other Allele*	*Effect Allele*	*EAF*	*Beta, SD*	*SE*	*P-value*	*Reported phenotype*	*Tested allele*	*Direction*	*Reference*
rs838133	*FGF21*	19	49259529	Sweet	A	G	0.57	-0.0325	0.0190	0.0866	Dietary macronutrient intake (protein)	A	-	Chu et al., 2013; PMID:23372041
				Sweet-fatty				-0.0361	0.0188	0.0551	Dietary macronutrient intake (carbohydrates)	A	+	Merino et al., 2018; PMID:29988085
				Naturally sweet items/fruits				0.0050	0.0189	0.7913	F-cake/biscuits liking (derived food-liking factor)	G	-	May-Wilson et al., 2022; PMID:35585065
				Sweeteners				-0.0072	0.0187	0.6990	Relative carbohydrate intake	A	+	Merino et al., 2022; PMID:34426670
rs7429279	*RARB*	3	25118637	Sweet	A	C	0.60	0.0204	0.0178	0.2520	F-sweet food liking (derived food-liking factor)	C	-	May-Wilson et al., 2022; PMID:35585065
				Sweet-fatty				0.0359	0.0177	0.0421	F-cake/biscuits liking (derived food-liking factor)	C	-	May-Wilson et al., 2022; PMID:35585065
				Naturally sweet items/fruits				0.0021	0.0177	0.9042				
				Sweeteners				-0.0102	0.0176	0.5623				
rs10889761	*COX6B1P7, ELOCP18**	1	68764949	Sweet	C	T	0.28	0.0345	0.0197	0.0803	Perceived intensity of sucrose	T	+	Hwang et al., 2019; PMID:31005972
				Sweet-fatty				0.0232	0.0196	0.2359				
				Naturally sweet items/fruits				0.0183	0.0197	0.3527				
				Sweeteners				0.0293	0.0194	0.1320				
rs35744813	*TAS1R3*	1	1265460	Sweet	T	C	0.94	-0.0511	0.0367	0.1635	Suprathreshold sensitivity to sucrose			Chamoun et al., 2021; PMID:33444969
				Sweet-fatty				-0.0425	0.0364	0.2429	Sucrose sensitivity scores	T	-	Fushan et al., 2009; PMID:19559618
				Naturally sweet items/fruits				-0.0153	0.0364	0.6744				
				Sweeteners				-0.0660	0.0363	0.0691				
rs10852521	*FTO*	16	53804965	Sweet	T	C	0.52	0.0391	0.0175	0.0255	Honey liking	C	+	May-Wilson et al., 2022; PMID:35585065
				Sweet-fatty				0.0363	0.0174	0.0369				
				Naturally sweet items/fruits				0.0323	0.0175	0.0650				
				Sweeteners				0.0327	0.0173	0.0584				

Salty modality

rsID	*Gene*	*Chr*	*Position*	*Tested phenotype*	*Other Allele*	*Effect Allele*	*EAF*	*Beta, SD*	*SE*	*P-value*	*Reported phenotype*	*Tested allele*	*Direction*	*Reference*
rs55872725	*FTO*	16	53809123	Salty-fatty	C	T	0.42	-0.0225	0.0176	0.1996	F-fatty/salty food liking (derived food-liking factor)	T	+	May-Wilson et al., 2022; PMID:35585065
rs72805612	*FTO*	16	53834608	Salty-fatty	G	A	0.43	-0.0161	0.0176	0.3607	Crisps liking	A	-	May-Wilson et al., 2022; PMID:35585065

Sour modality														
rsID	***Gene***	***Chr***	***Position***	***Tested phenotype***	***Other Allele***	***Effect Allele***	***EAF***	***Beta, SD***	***SE***	***P-value***	***Reported phenotype***	***Tested allele***	***Direction***	***Reference***
				Sour				0.0524	0.0176	0.0030				
rs2733520	*TBC1D5*	3	17246392	Sour-dairy	G	A	0.45	0.0430	0.0176	0.0144	F-fatty/dairy food liking (derived food-liking factor)	A	+	May-Wilson et al., 2022; PMID:35585065
				Sour-non-fatty				0.0416	0.0178	0.0194				
				Sour				0.0210	0.0180	0.2433				
rs2409764	*FAM167A-AS1, FAM167A*	8	11281273	Sour-dairy	A	G	0.45	0.0200	0.0178	0.2615	F-fatty/dairy food liking (derived food-liking factor)	G	+	May-Wilson et al., 2022; PMID:35585065
				Sour-non-fatty				0.0129	0.0181	0.4776				
				Sour				0.0015	0.0184	0.9370				
rs7652808	*CADM2*	3	85603643	Sour-dairy	T	G	0.67	0.0124	0.0183	0.4978	F-fatty/dairy food liking (derived food-liking factor)	G	-	May-Wilson et al., 2022; PMID:35585065
				Sour-non-fatty				-0.0038	0.0185	0.8371				
				Sour				0.0060	0.0199	0.7647				
rs710360	*LINC02144, TMEM161B**	5	87144026	Sour-dairy	C	G	0.26	-0.0173	0.0198	0.3844	F-dairy liking (derived food-liking factor)	G	-	May-Wilson et al., 2022; PMID:35585065
				Sour-non-fatty				0.0224	0.0201	0.2643				

Umami modality														
rsID	***Gene***	***Chr***	***Position***	***Tested phenotype***	***Other Allele***	***Effect Allele***	***EAF***	***Beta, SD***	***SE***	***P-value***	***Reported phenotype***	***Tested allele***	***Direction***	***Reference***
rs838133	*FGF21*	19	49259529	Umami				0.0196	0.0190	0.3025	Relative protein intake	A	-	Merino et al., 2022; PMID:34426670
				Umami-savory-meat	A	G	0.57	-0.0044	0.0187	0.8130	F-glutamate liking (derived food-liking factor)	G	+	May-Wilson et al., 2022; PMID:35585065
				Umami-other				0.0523	0.0193	0.0066	F-savoury food liking (derived food-liking factor)	G	+	May-Wilson et al., 2022; PMID:35585065
				Umami				-0.0405	0.0253	0.1100				
rs7256200	*APOC1*	19	45415935	Umami-savory-meat	G	T	0.14	-0.0303	0.0249	0.2239	F-glutamate liking (derived food-liking factor)	T	-	May-Wilson et al., 2022; PMID:35585065
				Umami-other				-0.0304	0.0256	0.2358				
				Umami				0.0046	0.0181	0.7982				
rs62250717	*CADM2*	3	85521990	Umami-savory-meat	C	G	0.39	-0.0033	0.0178	0.8521	Beef/steak liking	G	-	May-Wilson et al., 2022; PMID:35585065
				Umami-other				0.0159	0.0183	0.3850				
				Umami				-0.0231	0.0178	0.1930				
rs11642015	*FTO*	16	53802494	Umami-savory-meat	C	T	0.42	-0.0323	0.0175	0.0650	Salami liking	T	+	May-Wilson et al., 2022; PMID:35585065
				Umami-other				0.0108	0.0180	0.5499				
				Umami				0.0297	0.0191	0.1199				
rs9929436	*CDH13*	16	83661215	Umami-savory-meat	G	C	0.31	0.0159	0.0188	0.3971	Salami liking	C	+	May-Wilson et al., 2022; PMID:35585065
				Umami-other				0.0402	0.0193	0.0368				
				Umami				0.0276	0.0177	0.1186				
rs2733481	*TBC1D5*	3	17320397	Umami-savory-meat	G	A	0.45	0.0239	0.0174	0.1708	F-meat liking (derived food-liking factor)	A	+	May-Wilson et al., 2022; PMID:35585065
				Umami-other				0.0266	0.0179	0.1380				
				Umami				0.0175	0.0185	0.3451				
rs12629607	*CADM2*	3	85583608	Umami-savory-meat	T	C	0.67	0.0245	0.0182	0.1783	F-savoury food liking (derived food-liking factor)	C	-	May-Wilson et al., 2022; PMID:35585065
				Umami-other				0.0033	0.0187	0.8597				

Bitter-astringency modality														
rsID	***Gene***	***Chr***	***Position***	***Tested phenotype***	***Other Allele***	***Effect Allele***	***EAF***	***Beta, SD***	***SE***	***P-value***	***Reported phenotype***	***Tested allele***	***Direction***	***Reference***
rs1726866	*MGAM, TAS2R38*	7	141672705	Bitter-astringent	G	A	0.58	0.0236	0.0173	0.1705	Bitter taste perception (6-n-propylthiouracil) in obesity with metabolic syndrome	A	+	Coltell et al., 2019; PMID:31005965
				Bitter-astringent-beverages				-0.0122	0.0168	0.4687	Grapefruit liking	A	+	May-Wilson et al.,2022; PMID:35585065
				Bitter-astringent-others				0.0543	0.0177	0.0022				
rs1260326	*GCKR*	2	27730940	Bitter-astringent	T	C	0.63	0.0562	0.0177	0.0015	Caffeine consumption from coffee or tea	T	-	Said et al., 2020; PMID:33287642
				Bitter-astringent-beverages				0.0586	0.0173	0.0007	Bitter alcoholic beverage consumption	C	+	Zhong et al., 2019; PMID:31046077
				Bitter-astringent-others				0.0253	0.0182	0.1653	Bitter beverage consumption	C	+	Zhong et al., 2019; PMID:31046077
rs1229984	*ADH1B*	4	100239319	Bitter-astringent	T	C	0.98	0.1500	0.0687	0.0289	F-coffee/alcohol liking (derived food-liking factor)	C	+	May-Wilson et al., 2022; PMID:35585065
				Bitter-astringent-beverages				0.1454	0.0670	0.0300	Bitter alcoholic beverage consumption	C	+	Zhong et al., 2019; PMID:31046077
				Bitter-astringent-others				0.0650	0.0705	0.3564				
rs2472297	*CYP1A2, CYP1A1*	15	75027880	Bitter-astringent	C	T	0.29	0.0146	0.0186	0.4314	Bitter non-alcoholic beverage consumption	T	+	Zhong et al., 2019; PMID:31046077
				Bitter-astringent-beverages				0.0107	0.0181	0.5555	Bitter beverage consumption	T	+	Zhong et al., 2019; PMID:31046077
				Bitter-astringent-others				0.0152	0.0191	0.4254	Caffeine consumption from coffee	C	-	Said et al., 2020; PMID:33287642
rs504675	*LINC01833*	2	45154689	Bitter-astringent	C	T	0.37	0.0170	0.0178	0.3394	F-bitter food liking (derived food-liking factor)	T	+	May-Wilson et al., 2022; PMID:35585065
				Bitter-astringent-beverages				0.0116	0.0174	0.5055				
				Bitter-astringent-others				0.0148	0.0183	0.4171				
rs7619139	*RARB*	3	25110415	Bitter-astringent	T	A	0.60	0.0092	0.0172	0.5930	Raw vegetable consumption	T	-	Cole et al., 2020; PMID:32193382
				Bitter-astringent-beverages				0.0018	0.0168	0.9144	F-salad vegetables liking (derived food-liking factor)	A	+	May-Wilson et al., 2022; PMID:35585065
				Bitter-astringent-others				0.0129	0.0177	0.4654	Cabbage liking	A	+	May-Wilson et al., 2022; PMID:35585065

Pungency modality														
rsID	***Gene***	***Chr***	***Position***	***Tested phenotype***	***Other Allele***	***Effect Allele***	***EAF***	***Beta, SD***	***SE***	***P-value***	***Reported phenotype***	***Tested allele***	***Direction***	***Reference***
rs224537	*TRPV1*	17	3487202	Pungent	G	A	0.38	-0.0045	0.0181	0.8026	Spicy food liking	A	+	May-Wilson et al., 2022; PMID:35585065
rs504675	*LINC01833*	2	45154689	Pungent	C	T	0.37	0.0302	0.0183	0.0992	F-burn/spicy liking (derived food-liking factor)	T	+	May-Wilson et al., 2022; PMID:35585065
rs2029130	*CADM2*	3	85644047	Pungent	T	A	0.65	0.0065	0.0184	0.7222	Spicy food liking	A	-	May-Wilson et al., 2022; PMID:35585065
rs4939727	*DCC*	18	50792914	Pungent	G	A	0.38	0.0360	0.0180	0.0452	F-capsicum liking (derived food-liking factor)	A	+	May-Wilson et al., 2022; PMID:35585065
rs11859365	*CDH13*	16	83683945	Pungent	A	C	0.23	0.0207	0.0207	0.3162	F-sharp flavour liking (derived food-liking factor)	C	+	May-Wilson et al., 2022; PMID:35585065
rs9257566	*OR2J2, OR2J4P**	6	29144532	Pungent	C	T	0.12	-0.0435	0.0282	0.1222	Horseradish liking	T	-	May-Wilson et al., 2022; PMID:35585065

Results for the tested phenotypes are obtained with the rank-based inverse normal transformed liking scores and adjusted for age, age^2 and sex. The reported phenotypes in the literature with the tested allele and direction of effect are listed for each candidate SNP. Chr, chromosome; Beta, effect size; SD, standard deviation; EAF, effect allele frequency; SE, standard error; * Nearest upstream and downstream genes; Genomic locations are shown as GRCh37/hg19.

Supplementary Table 6. Pathway enrichment analysis in GeneNetwork

Pathway name	P-value	Database
Transmission across Chemical Synapses	4.97E-07	REACTOME
Neurotransmitter receptors and postsynaptic signal transmission	1.41E-06	REACTOME
Metabolism of RNA	1.47E-06	REACTOME
Platelet homeostasis	2.75E-06	REACTOME
Regulation of commissural axon pathfinding by SLIT and ROBO	5.18E-06	REACTOME
Effects of PIP2 hydrolysis	5.19E-06	REACTOME
Collagen chain trimerization	2.34E-05	REACTOME
Neuronal System	3.25E-05	REACTOME
Translation	3.28E-05	REACTOME
Ca2+ pathway	5.74E-05	REACTOME
rRNA processing	7.86E-05	REACTOME
GABA synthesis, release, reuptake and degradation	8.20E-05	REACTOME
Ion channel transport	9.19E-05	REACTOME
Olfactory Signaling Pathway	1.06E-04	REACTOME
EPH-Ephrin signaling	1.15E-04	REACTOME
Semaphorin interactions	1.18E-04	REACTOME
NCAM signaling for neurite out-growth	1.26E-04	REACTOME
Collagen biosynthesis and modifying enzymes	1.31E-04	REACTOME
NCAM1 interactions	1.47E-04	REACTOME
Ion transport by P-type ATPases	1.62E-04	REACTOME
Axon guidance	1.28E-04	KEGG
Olfactory transduction	1.47E-04	KEGG
Gap junction	1.65E-04	KEGG
Arrhythmogenic right ventricular cardiomyopathy (ARVC)	1.47E-03	KEGG
Mapk signaling pathway	3.66E-03	KEGG
Calcium signaling pathway	5.78E-03	KEGG

Neurotrophin signaling pathway	6.74E-03	KEGG
Arachidonic acid metabolism	8.44E-03	KEGG
Spliceosome	8.54E-03	KEGG
Glioma	9.44E-03	KEGG
Wnt signaling pathway	9.93E-03	KEGG
Chemokine signaling pathway	1.05E-02	KEGG
Endocytosis	1.06E-02	KEGG
Focal adhesion	1.13E-02	KEGG
Pathways in cancer	1.31E-02	KEGG
Small cell lung cancer	1.40E-02	KEGG
Linoleic acid metabolism	1.75E-02	KEGG
Phosphatidylinositol signaling system	2.07E-02	KEGG
Mtor signaling pathway	2.12E-02	KEGG
Aldosterone regulated sodium reabsorption	2.15E-02	KEGG
neuron migration	1.52E-06	GO Biological Process
detection of chemical stimulus involved in sensory perception of smell	2.84E-06	GO Biological Process
central nervous system development	3.36E-06	GO Biological Process
axon guidance	1.12E-05	GO Biological Process
dendrite morphogenesis	1.30E-05	GO Biological Process
response to nicotine	1.34E-05	GO Biological Process
regulation of signal transduction by p53 class mediator	2.84E-05	GO Biological Process
embryonic digit morphogenesis	4.23E-05	GO Biological Process
regulation of GTPase activity	4.31E-05	GO Biological Process
peripheral nervous system development	5.14E-05	GO Biological Process
sensory perception of sound	5.46E-05	GO Biological Process
cAMP-mediated signaling	7.07E-05	GO Biological Process
ion transmembrane transport	7.41E-05	GO Biological Process
axonogenesis	7.73E-05	GO Biological Process
signal transduction	8.34E-05	GO Biological Process
calcium ion transmembrane transport	9.30E-05	GO Biological Process

ephrin receptor signaling pathway	1.00E-04	GO Biological Process
positive regulation of GTPase activity	1.02E-04	GO Biological Process
intracellular signal transduction	1.07E-04	GO Biological Process
synapse assembly	1.38E-04	GO Biological Process
ion channel binding	1.52E-06	GO Molecular Function
olfactory receptor activity	3.96E-06	GO Molecular Function
beta-catenin binding	8.39E-06	GO Molecular Function
RNA binding	9.59E-06	GO Molecular Function
rRNA binding	5.24E-05	GO Molecular Function
calmodulin binding	7.86E-05	GO Molecular Function
14-3-3 protein binding	1.11E-04	GO Molecular Function
ARF guanyl-nucleotide exchange factor activity	1.42E-04	GO Molecular Function
odorant binding	1.45E-04	GO Molecular Function
signal transducer activity	1.58E-04	GO Molecular Function
PDZ domain binding	1.59E-04	GO Molecular Function
calcium ion binding	1.69E-04	GO Molecular Function
calcium channel activity	2.07E-04	GO Molecular Function
E-box binding	3.63E-04	GO Molecular Function
bitter taste receptor activity	4.04E-04	GO Molecular Function
Ras guanyl-nucleotide exchange factor activity	4.17E-04	GO Molecular Function
RNA polymerase II activating transcription factor binding	5.77E-04	GO Molecular Function
GTPase activator activity	5.86E-04	GO Molecular Function
ligand-gated ion channel activity	8.28E-04	GO Molecular Function
Wnt-protein binding	9.47E-04	GO Molecular Function
dendrite	4.64E-07	GO Cellular Component
axon	1.11E-06	GO Cellular Component
postsynaptic membrane	4.36E-06	GO Cellular Component
presynaptic membrane	8.90E-06	GO Cellular Component
synapse	1.32E-05	GO Cellular Component
dendritic spine	6.71E-05	GO Cellular Component

receptor complex	7.81E-05	GO Cellular Component
postsynaptic density	8.35E-05	GO Cellular Component
plasma membrane	9.69E-05	GO Cellular Component
neuron projection	1.40E-04	GO Cellular Component
growth cone	1.47E-04	GO Cellular Component
neuronal cell body	2.10E-04	GO Cellular Component
filopodium	3.14E-04	GO Cellular Component
nucleolus	3.94E-04	GO Cellular Component
apical plasma membrane	4.84E-04	GO Cellular Component
perikaryon	4.87E-04	GO Cellular Component
clathrin-coated vesicle	5.50E-04	GO Cellular Component
cell junction	6.48E-04	GO Cellular Component
Golgi apparatus	6.92E-04	GO Cellular Component
cell	9.20E-04	GO Cellular Component

Results are based on a gene set of 169 out of 171 overlapped genes with SNPs suggestively associated with the 6 modalities and underlying factors of the TasteLQ. The top 20 significant pathways for the gene set are shown for each database (REACTOME, KEGG, GO). Statistical significance assessed using Wilcoxon test as reported by GeneNetwork v2.0. GO.; Gene ontology; KEGG; Kyoto Encyclopedia of Genes and Genome.

Supplementary References

1. Costa MM, Da Silva AP, Santos C, Ferreira J, Mascarenhas MR, Bicho M, et al. Influence of the TAS2R38 Gene Single Nucleotide Polymorphisms in Metabolism and Anthropometry in Thyroid Dysfunction. Nutrients. 2023 May 6;15(9).

2. Haydar S, Karlsson CC, Linneberg A, Kårhus LL, Ängquist L, Hansen T, et al. The Taste Liking Questionnaire (TasteLQ) – A tool for assessment of taste liking in the Danish population. Food Quality and Preference. 2024 Aug;117:105176.
